# Somatic embryogenesis receptor-like kinase 5 in the ecotype Landsberg *erecta* of *Arabidopsis* is a functional RD LRR-RLK in regulating brassinosteroid signaling and cell death control

**DOI:** 10.3389/fpls.2015.00852

**Published:** 2015-10-15

**Authors:** Wangze Wu, Yujun Wu, Yang Gao, Meizhen Li, Hongju Yin, Minghui Lv, Jianxin Zhao, Jia Li, Kai He

**Affiliations:** ^1^Ministry of Education Key Laboratory of Cell Activities and Stress Adaptations, School of Life Sciences, Lanzhou UniversityLanzhou, China; ^2^Crop Research Institute, Anhui Academy of Agricultural SciencesHefei, China

**Keywords:** *Arabidopsis*, brassinosteroid, cell death, LRR-RLK, natural variation, RD kinase, SERK, signal transduction

## Abstract

In plants, LRR-RLKs play central roles in regulating perception of extracellular signals and initiation of cellular responses under various environmental challenges. *Arabidopsis SERK* genes, including *SERK1* to *SERK5*, constitute a *LRR*-*RLK* sub-family. *SERK1, SERK2, SERK3*/*BAK1*, and *SERK4*/*BKK1* have been well characterized to function as crucial regulators in multiple physiological processes such as brassinosteroid signaling, cell death control, pathogenesis, and pollen development. Despite extremely high sequence identity with *BKK1, SERK5* is reported to have no functional overlapping with *BKK1*, which is previously identified to regulate BR and cell death control pathways, probably due to a natural mutation in a highly conserved RD motif in the kinase domain of SERK5 in Col-0 ecotype. Through a gene sequencing analysis in several *Arabidopsis* accessions, we are able to identify *SERK5* in Landsberg *erecta* (L*er*) genome encoding a LRR-RLK with an intact RD motif. Overexpression of *SERK5*-L*er* partially suppresses the BR defective phenotypes of *bri1-5* and *bak1-3 bkk1-1*, indicating *SERK5*-L*er* functions as a positive regulator in BR signaling. Furthermore, the interaction between SERK5-L*er* and BRI1 is confirmed by yeast two-hybrid and BiFC assays, and the genetic result showing that elevated expression of a kinase-dead form of *SERK5*-L*er* causes a dominant-negative phenotype in *bri1-5*. In addition, overexpression of *SERK5*-L*er* is capable of delaying, not completely suppressing, the cell death phenotype of *bak1-3 bkk1-1*. In this study, we first reveal that *SERK5*-L*er* is a biologically functional component in mediating multiple signaling pathways.

## Introduction

During evolution, plants have developed multiple strategies to sense surrounding cues in order to adapt to the environment. Perception of extracellular stimuli and initiation of intracellular signaling cascades serve as key processes for plant cells to make different responses under normal and various stressed conditions. Due to their unique structural properties, receptor-like protein kinases (RLKs) in plants play essential roles in cell-cell and cell-environment communications (Li, 2010). RLKs contain an extracellular domain and a cytosolic kinase domain, connected via a single-pass transmembrane helix. The ectodomains of RLKs perceive the apoplastic signals including phytohormones and small peptides followed by the activation of cytosolic kinase domain triggering protein phosphorylation cascades as responses. In *Arabidopsis*, there are more than 610 RLKs, including about 135 receptor-like cytoplasmic kinases (RLCKs), which lack the ectodomains (Shiu and Bleecker, 2001a). As the largest RLK subfamily, leucine-rich repeat RLK (LRR-RLK) family contain at least 223 members, featured by 1–32 copies of leucine-rich repeats in their extracellular domains (Shiu and Bleecker, 2001a,b; Lehti-Shiu et al., 2009). LRR-RLKs are grouped into 13 subfamilies (LRR I to XIII), based on their protein sequence similarity in their kinase domains (Shiu and Bleecker, 2001a). In the kinase subdomain VIb of a number of LRR-RLKs, an Arg (R) residue resides preceding a highly conserved Asp (D) residue, referred to an RD motif, followed by the activation loop. Arg (R) residue is sometimes replaced by uncharged residues such as Leu (L), Gly (G), Cys (C), or Phe (F) in some LRR-RLKs (Krupa et al., 2004). Thus, LRR-RLKs can also be categorized into RD kinases and non-RD kinases according to the presence or absence of an RD motif in their kinase domains. For most RD LRR-RLKs, the phosphorylation in the activation loop is crucial for triggering kinase activity which usually displays high (auto- and trans-) phosphorylation ability. Non-RD LRR-RLKs do not require phosphorylating the activation loop and usually exhibit lower kinase activities (Johnson et al., 1996; Nolen et al., 2004; Schwessinger et al., 2011). Distinct molecular mechanisms contribute to non-RD kinase activation, such as keeping constitutively active, release of C-terminus to remove auto-inhibition, or Tyr (Y) phosphorylation in kinase domain (Kobe et al., 1996; Mayans et al., 1998; Nolen et al., 2001).

One well-established model for paired LRR-RLK receptors functioning in signal sensing and transduction includes brassinosteroid (BR) receptor BRASSINOSTEROID INSENSITIVE 1 (BRI1) (Li and Chory, 1997) and its co-receptor BRI1-ASSOCIATED KINASE 1 (BAK1) (Li et al., 2002; Nam and Li, 2002). Genetics, biochemistry, and protein structure analyses have demonstrated that BRI1 is an indispensable bona fide receptor of brassinolide (BL), the most biologically active form of BR (He et al., 2000; Wang et al., 2001; Kinoshita et al., 2005; Hothorn et al., 2011; She et al., 2011). BAK1 was identified as an interacting protein of BRI1 (Li et al., 2002; Nam and Li, 2002), acting as a co-receptor of BRI1 (Li, 2010). Recent genetic and protein structure results support the irreplaceable role of BAK1 and its homologs in BR signaling via a direct association with a BL molecule (Gou et al., 2012; Santiago et al., 2013; Sun et al., 2013b). Upon BR perception, the BRI1-BAK1 complex initiates downstream signaling cascades and ultimately finely tunes the transcriptional programs to regulate various aspects of plant development, including cell expansion and division, vascular differentiation, cell proliferation, male fertility, senescence, induction of flowering, and stress responses (Clouse and Sasse, 1998; He et al., 2001; Nakaya et al., 2002; Symons et al., 2006; Divi and Krishna, 2009; Ye et al., 2010; Belkhadir and Jaillais, 2015).

BAK1 belongs to SOMATIC-EMBRYOGENESIS RECEPTOR-LIKE KINASE (SERK) sub-family with five members (SERK1 to SERK5) (Hecht et al., 2001). BAK1 is also known as SERK3, and SERK4, the closest homolog of BAK1, was named as BAK1-Like 1 (BKK1) (He et al., 2007). In *Arabidopsis, SERKs* participate in multiple signaling pathways in which they are often functionally redundant (He et al., 2007; Albrecht et al., 2008; Roux et al., 2011; Schwessinger et al., 2011; Du et al., 2012; Gou et al., 2012). In addition to BAK1, SERK1/2 and BKK1 were also discovered to interact with BRI1, functioning as positive regulators of BR responses (Karlova et al., 2006; He et al., 2007; Albrecht et al., 2008; Gou et al., 2012; Santiago et al., 2013).

Intriguingly, our previous studies revealed a novel function of *BAK1* and *BKK1* in a BR-independent cell death control pathway (He et al., 2007). *bak1-4 bkk1-1*, a null double mutant, shows a spontaneous cell death phenotype within a week after germination even on the sterilized medium (He et al., 2007). BAK1 was also found to associate with FLAGELLIN SENSITIVE 2 (FLS2), Elongation Factor-Tu Receptor 1 (EFR1) and BAK1-interacting receptor-like kinase 1 (BIR1), respectively, to regulate innate immunity (Chinchilla et al., 2007; Heese et al., 2007; Gao et al., 2009; Schulze et al., 2010; Sun et al., 2013a). BAK1, therefore, has been proposed to be an adaptor protein required for various ligand-binding RLKs to perceive diverse signal molecules and to initiate corresponding downstream signaling pathways (He et al., 2013).

*BKK1* is the closed paralog of *BAK1* and plays a redundant role with *BAK1* at least in BR and cell death control pathways. The closest paralog of *BKK1* (At2G13790) is *SERK5* (At2G13800) and they exist as a pair of tandem-repeated genes on chromosome 2 (He et al., 2007). SERK5 shares 85% amino acids identity with BKK1, suggesting BKK1 and SERK5 are very likely functionally redundant. However, no reports to date have demonstrated *SERK5* is involved in regulating BR signaling or cell death control (He et al., 2007; Albrecht et al., 2008; Jeong et al., 2010; Roux et al., 2011; Gou et al., 2012). Our previous studies showed that overexpression of *BKK1*, not *SERK5*, is able to partially suppress *bri1-5*, a weak *BRI1* mutant allele (He et al., 2007; Gou et al., 2012). By using coimmunoprecipitation and mass spectrometry analyses, it has been shown that EFR and FLS2 form heteromerization, in a ligand-induced manner, with SERK1, SERK2, BAK1 and BKK1 but not SERK5 (Roux et al., 2011).

Detailed sequencing analysis indicates that SERK5 in Col-0 ecotype, which was used in previous studies, bears an amino acid substitution of Leu (L) for Arg (R) within the RD motif in the kinase domain, and this substitution might abolish the function of Col-0 SERK5 in the BR signaling pathway (He et al., 2007). It remains to be determined that whether SERK5 containing an intact RD motif exists in other *Arabidopsis* ecotypes and whether it is functionally active.

In this study, we aimed to elucidate the biological functions of *SERK5*. To this end, we analyzed *SERK5* sequences in five *Arabidopsis* accessions. Interesting enough, *SERK5* in the genome of Landsberg *erecta* (Ler) ecotype was identified to encode a LRR-RLK with a normal RD motif. By using genetic, biochemistry and molecular biology approaches, *SERK5*-L*er* has been analyzed in BR signaling as a functional regulator. Overexpression of *SERK5*-L*er* was capable of delaying cell death phenotype of *bak1-3 bkk1-1*, a weak double mutant, suggesting *SERK5*-L*er* also plays a role in cell death control.

To date, little is yet known about the functional roles of *SERK5* in *Arabidopsis*. Our results first unfold *SERK5* in an *Arabidopsis* accession as a biologically functional mediator in regulating BR and cell death pathways, similar to its closest paralog, *BKK1*.

## Materials and methods

### Materials and plant growth conditions

*Arabidopsis thaliana* ecotypes Landsberg *erecta* (L*er*), Columbia-0 (Col-0), Wassilewskija-2 (Ws-2), Lanark-0 (Lan-0) and C24 were used in this study. Plants were grown at 22°C under 16 h light/8 h dark, unless otherwise specified. For BR sensitivity assays, seedlings were grown on 1/2 MS medium supplemented with 1% sucrose, 0.8% agar and various concentrations of 24-epiBL (Sigma, St. Louis, MO).

### Gene cloning and *Arabidopsis* transformation

Full-length cDNAs and genomic DNA fragments of *SERK5* in five ecotypes were cloned into expression binary vector pB35GWF by a gateway strategy as described in previous study (Gou et al., 2012). Site-directed mutagenesis was performed according to the manual of the QuickChange Site-Directed Mutagenesis Kit (Stratagene, La Jolla, CA). All of the cloned DNA sequences were confirmed by sequencing analysis. The cloned genes were transformed into *Arabidopsis* plants by a floral dip method (Clough and Bent, 1998). T_1_ seedlings were screened using 0.1% (v/v) basta in the soil. All the primers used for cloning are listed in Table S1.

### Determination of plant fresh weight and leaf area

Thirty five-day-old seedlings grown in the greenhouse were weighted and the data were analyzed by SPSS 15.0 software (http://www-01.ibm.com/software/analytics/spss/). For leaf area measurement, fully expanded leaves of 30-day-old seedlings grown in the greenhouse were striped and imaged. The areas of the leaves were determined using Image-Pro Plus software (http://www.mediacy.com/index.aspx?page=IPP). All measurements were repeated at least three times. About 30–60 seedlings were measured each time. All data were presented as the mean ± standard deviation (SD). Student's *t*-test was used to show statistical differences.

### Root and hypocotyl growth analyses

The seeds planted on 1/2 MS medium were vernalizated at 4°C for 2 days. Seedlings were grown vertically in continuous light or dark at 22°C. The root lengths were measured 8 days after germination in the light and the hypocotyl lengths were measured 8 days after germination in the dark.

### Gene expression analyses

Total RNA was extracted from the seedlings according to the manual of the RNAprep Pure Plant Kit [TIANGEN BIOTECH (BEIJING) CO., LTD]. For semi-quantitative RT-PCR to confirm overexpression of *SERKs* in the transgenic plants, RNA was isolated from the leaves of 4-week-old plants grown in the soil. For BR sensitivity test, total RNA was isolated from 10-day-old seedlings grown on 1/2 MS medium treated with or without 1 μM 24-epiBL for 3 h. For the analysis of expression levels of defense- and senescence-related genes, total RNA was isolated from the 13-day-old seedlings grown on 1/2 MS medium. Two micrograms of total RNA was reversely transcribed in a 20 μl volume using Superscript III reverse transcriptase (Invitrogen). The transcripts of specific genes were quantitatively analyzed with SYBR Premix ExTaq kit (TaKaRa). *ACTIN2* was used as an internal reference. The primers used for semi-quantitative RT-PCR and qRT-PCR are listed in Table S1.

### Protein extraction and western analysis

Total protein was extracted from 3-week-old plants as described by Wang et al. (2005). Protein samples were separated on 10% (w/v) SDS-PAGE gel. Western blotting using α-FLAG antibodies (Sigma, St. Louis, MO) were performed as previously described (Li et al., 2002).

### Trypan-blue staining

The leaves from 10 to16-day-old plants were incubated in trypan-blue staining solution (8 ml of 95% ethanol, 1 ml of ddH_2_O, 1 ml of lactic acid, 1 ml of glycerol, 1 ml of phenol, 0.2 mg trypan-blue) in 1.5 ml tubes and boiled in a water bath for 2–3 min. After cooling to room temperature, the solution was replaced with chloral hydrate (2.5 g/ml). The chloral hydrate solution was replaced a few times until the leaves were fully destained.

### Mating-based split ubiquitin system

The mating-based split ubiquitin system (mbSUS) method (Obrdlik et al., 2004) was utilized to test the interaction between BRI1 and SERKs. The full-length *BRI1* was introduced into pMetYCgate vector (bait vector) by gateway cloning technology and transformed into the yeast THY.AP4 (Cubs) strain. The full-length *SERK5*-L*er, SERK5*, and *BKK1* were introduced into pX-NubWTgate vector (prey vector) by gateway cloning technology and transformed into the yeast THY.AP5 (Nubs) strain. *BRI1* was also introduced into pX-NubWTgate vector, wherein *SERK5*-L*er, SERK5* and *BKK1* were introduced into pMetYCgate vector, followed by being transformed into the yeast THY.AP5 (Nubs) strain and THY.AP4 (Cubs) strain, respectively. Yeast-diploid colonies were obtained after mating and selected on the selection medium. The protein-protein interaction was tested by growing yeast on the synthetic minimal medium lacking adenine, histidine, leucine, and tryptophan (-AHLT) and containing 0, 75, 150, and 400 μM methionine. Interactions were also verified using β-galactosidase assays as previously described (Obrdlik et al., 2004). The strength of interaction was quantified by measuring the Integrated Optical Density (IOD) of the growing yeast using Image-Pro Plus software (http://www.mediacy.com/index.aspx?page=IPP).

### Bimolecular fluorescence complementation (BIFC) assay

The full-length cDNA of *BRI1* was cloned into vector pEG-GWR-nYFP (*BRI1*-*nYFP*), and the full-length cDNAs of *SERK5*-L*er, BKK1* and *SERK5* were cloned into vector pEG-GWR-cYFP (*SERK5*-L*er*-*cYFP, SERK5*-*cYFP*, and *BKK1*-*cYFP*). The above constructs were transformed into *Agrobacterium tumefaciens* strain GV3101 using electroporation. The transformed GV3101 cells were cultured overnight in LB medium containing 20 μM acetosyringone. Re-suspended the cultures to OD_600 nm_ = 0.5 with injection buffer (liquid MS medium containing 150 μM acetosyringone, 10 mM MgCl_2_, 10 mM MES, pH 5.7). *BRI*-*nYFP* and *SERK5*-L*er*/*SERK5*/*BKK1*-*cYFP* cultures were mixed by equal volumes. The mixtures were incubated for at least 2 h at room temperature, and were co-injected into the leaves of 3-week-old *Nicotiana benthamiana*. After 72 h, YFP fluorescence emitted from the epidermal cells of the injected leaves was observed with confocal laser scanning microscopy (Olympus FV1000-IX81).

## Results

### Landsberg *erecta* (L*er*) SERK5 is an RD LRR-RLK

**Figure 1:**
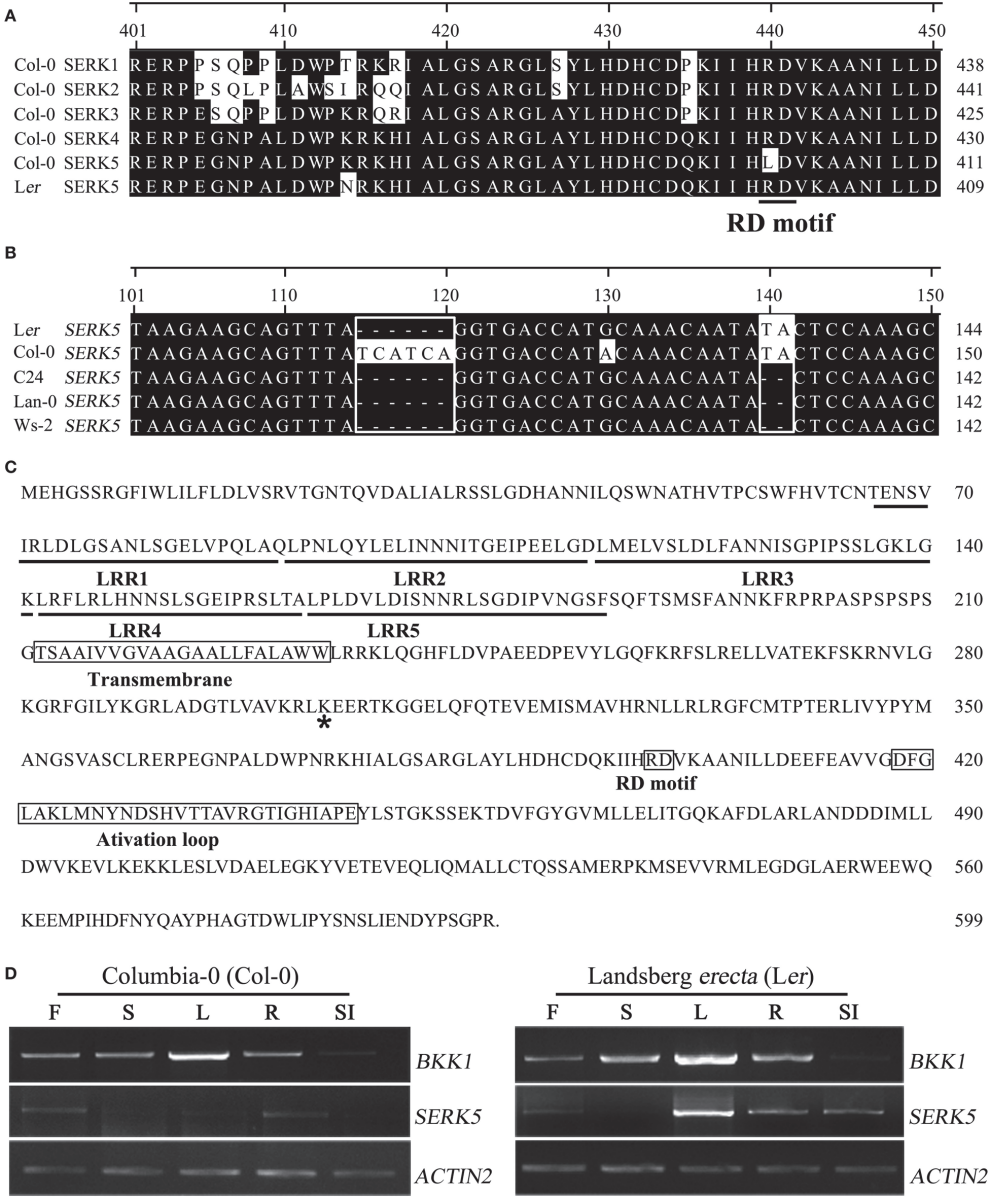
*****SERK5*** in Landsberg ***erecta*** (L***er***) ecotype encodes a LRR-RLK with a normal RD motif**. **(A)** Amino acid sequence alignment (position 401–450) of SERK1-5 in Col-0 ecotype and SERK5 in L*er* ecotype. SERK5-L*er* contains a normal RD motif. R is substituted by L at position 401 in SERK5-Col. **(B)** cDNA sequence alignment (position 101–150) of *SERK5* in L*er*, Col-0, C24, Lan-0, and Ws-2. *SERK5* in Col-0 contains extra six nucleotides in alignment position 115–120. *SERK5* in C24, Lan-0, and Ws-2 contains two-nucleotide (TA) deletion at alignment position 140–141. **(C)** Full protein sequence of SERK5 in L*er*. Asterisk mark indicates the conserved lysine (K) in ATP binding domain. **(D)** Expression levels of *BKK1* and *SERK5* in Col-0 (left) and Landsberg *erecta* (L*er*) (right) ecotype are shown in all plant tissue. Semi-quantitative RT-PCR was carried out with RNA isolated from the 4 weeks old Col-0 and L*er* ecotype *Arabidopsis* grown in soil. The PCR amplification cycles for *BKK1, SERK5* and quantitative control *ACTIN2*, are 30, 30, and 19, respectively. F, flowers; S, stems; L, rosette leaves; R, roots; SI, siliques.

Gain-of-function and loss-of-function genetic analyses have demonstrated that *SERK1, SERK2, SERK3*/*BAK1*, and *SERK4*/*BKK1*, but not *SERK5*, in Col-0 ecotype regulate BR signaling (Karlova et al., 2006; He et al., 2007; Albrecht et al., 2008; Gou et al., 2012). Considering that BKK1 and SERK5 share extremely high protein sequence identity (85%), it is puzzling that *SERK5* exhibits no functional redundancy with *BKK1*. This may be explicated by detailed sequence analysis showing that SERK5 in Col-0 contains a substitution of R by L in RD motif, which is different from BKK1, a canonical RD kinase (He et al., 2007). We thus speculated that the eradication of RD motif in SERK5 may abolish its biological functions in Col-0 ecotype. We started analyzing *SERK5* sequences in different *Arabidopsis* accessions. *SERK5* cDNAs from Landsberg *erecta* (L*er*), Wassilewskija-2 (Ws-2), Columbia-0 (Col-0), Lanark-0 (Lan-0), and C24 ecotypes were cloned using a gateway strategy. Sequence alignment results indicated that SERK5 in L*er*, Ws-2, Lan-0, or C24, but not in Col-0, contains an RD motif (Figure 1A). Unexpectedly, the cDNA sequences of *SERK5* from C24, Lan-0 and Ws-2 contains a two-nucleotide base (TA) deletion (Figure 1B). As a result, the *SERK5* in C24, Lan-0 and Ws-2 is not able to be properly translated, due to a shifted open reading frame. Furthermore, *SERK5* in ecotype C24, Lan-0 and Ws-2 and L*er* has a six-nucleotide deletion, leading to absence of two Ser (S) residues (Figure 1B). In order to further verify the *SERK5* cDNAs, genomic DNA fragments of *SERK5* in five ecotypes were cloned. Sequencing results confirmed that only *SERK5* genomic DNA sequence in L*er* has no TA deletion and maintains a typical RD motif (data not shown). The full-length CDS of *SERK5* in L*er* is 1800 bp, encoding a protein of 599 amino acids (Figure 1C), showing 98% and 85% similarity to SERK5 and BKK1 in Col-0, respectively. Given that SERK5 in L*er* is truly an RD LRR-RLK, it is still essential to show *SERK5* is indeed expressed in L*er* ecotype if one claims *SERK5* in L*er* is physiologically active. By using RT-PCR assay with same cycles (30 cycles), we analyzed the expression patterns of *SERK5* in various tissues in both Col-0 and L*er* backgrounds, starting with equally amount of total RNA. Our results suggested that the expression levels of *SERK5* are much lower in Col-0 than those in L*er* (Figure 1D). By contrast, *BKK1* was highly expressed in almost all tissues and exhibited the same expression patterns in Col-0 and L*er*. Interestingly, compared to *SERK5* in Col-0, the transcripts of *SERK5* in L*er* were significantly higher, especially in rosette leaves where *BKK1* also showed the highest expression level (Figure 1D). These results indicated that *SERK5* is substantially expressed in L*er* with markedly higher expression levels than in Col-0 in which *SERK5* is inactive. Taken together, SERK5 in L*er* ecotype was identified as a typical RD LRR-RLK, which is subsequently named as SERK5-L*er*. In this article, all *SERKs* refer to those in Col-0 ecotype unless being specifically designated.

### Overexpressing *SERK5*-L*er* can partially suppress *bri1*-5 phenotype

**Figure 2:**
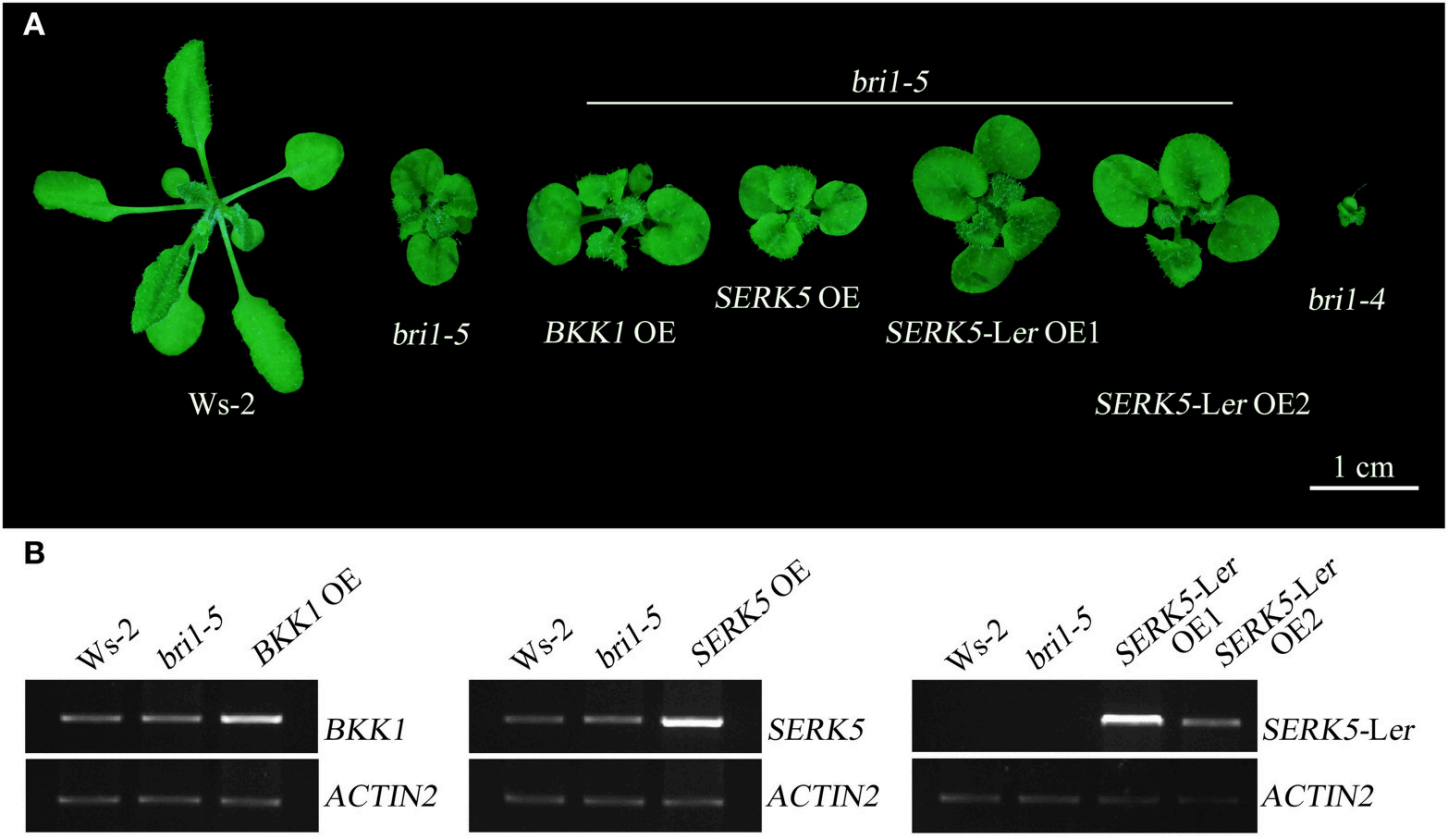
**Overexpressing ***SERK5***-L***er*** can partially suppress ***bri1-5*** phenotype**. **(A)** Overexpression of *SERK5*-L*er* and *BKK1*, but not *SERK5*, can partially suppresses the phenotypes of *bri1-5*, a weak *BRI1* mutant. *bri1-4* is a null *BRI1* mutant allele. **(B)** RT-PCR analyses to confirm the elevated expression of the transgenes in *bri1-5*. The PCR amplification cycles for *BKK1, SERK5, SERK5*-L*er* and quantitative control *ACTIN2*, are 28, 28, 27, and 19, respectively.

**Figure 3:**
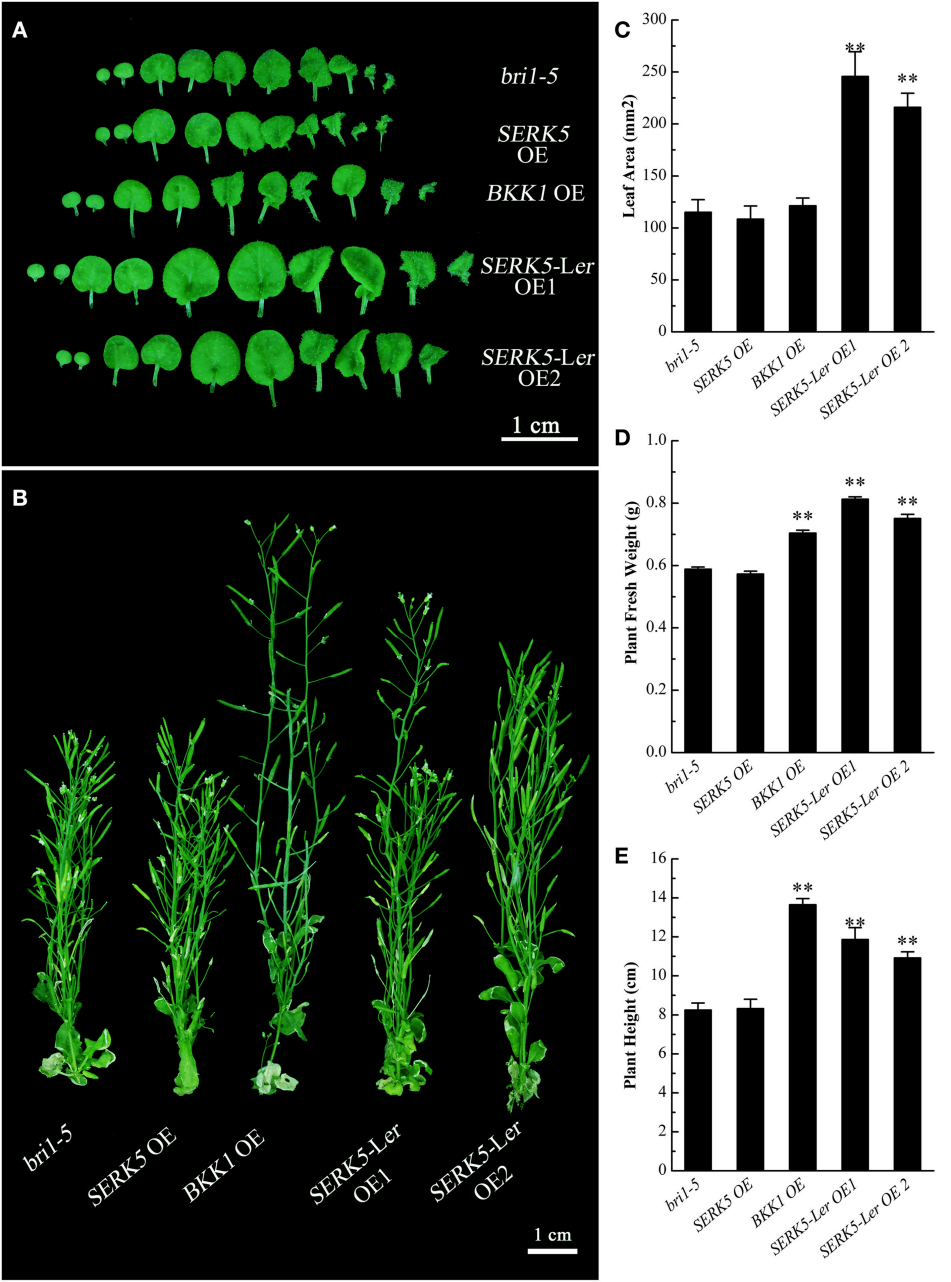
**Detailed phenotypic analyses of ***bri1-5*** overexpressing ***SERKs*****. **(A)** Overexpression of *SERK5*-L*er*, but not *BKK1* and *SERK5*, enlarges the leaf size of *bri1-5*. **(B)** Overexpression of *SERK5*-L*er* and *BKK1*, but not and *SERK5*, increases the plant height of *bri1-5*. **(C)** Statistic analyses of leaf areas of the plants presented in **(A)**. **(D)** Statistic analyses of plant fresh weight of 35-day-old seedlings. **(E)** Statistic analyses of plant heights presented in **(B)**. The data are shown as means ± standard deviation (SD) (*n* = 20). Student's *t*-test indicated the differences are statistically significant (^**^P < 0.01). Experiments were repeated three times with similar results.

We have shown in the earlier reports that overexpression of *SERK1* to *SERK4* can partially suppress the BR defective phenotype of *bri1-5* (He et al., 2007; Gou et al., 2012). We speculated that SERK5-L*er*, possessing a normal RD motif, may share similar biological functions with other SERK members in BR signaling. To test this hypothesis, we used the *CaMV35S* promoter to drive *SERK5*-L*er* in *bri1-5*. Two overexpression transgenic lines, named as *SERK5*-L*er* OE1 and *SERK5*-L*er* OE2, were confirmed by RT-PCR and western-blotting (Figure 2B, Figure S1A). Indeed, elevated expression of *SERK5*-L*er* or *BKK1*, but not *SERK5*, was able to partially suppress *bri1-5*. Overexpressing *SERK5*-L*er* dramatically enlarged the leaf size of *bri1-5*, which was not observed in *bri1-5* lines overexpressing *BKK1* (Figures 3A,C). Overexpression of *BKK1* was capable of suppressing the phenotypes of shortened petioles in *bri1-5*, while enhanced expression of *SERK5*-L*er* failed to do so (Figure S2A). This result indicated that *BKK1* and *SERK5*-L*er* may play distinct roles in BR signaling during different developmental stages or in diverse plant tissues. Overexpression of either *BKK1* or *SERK5*-L*er* in *bri-5* also noticeably increased the plant height compared to *bri-5* background at 55 days (Figures 3B,E). Overexpression of *SERK5*-L*er* or *BKK1* also increased *bri1-5* fresh weight (Figure 3D).

**Figure 4:**
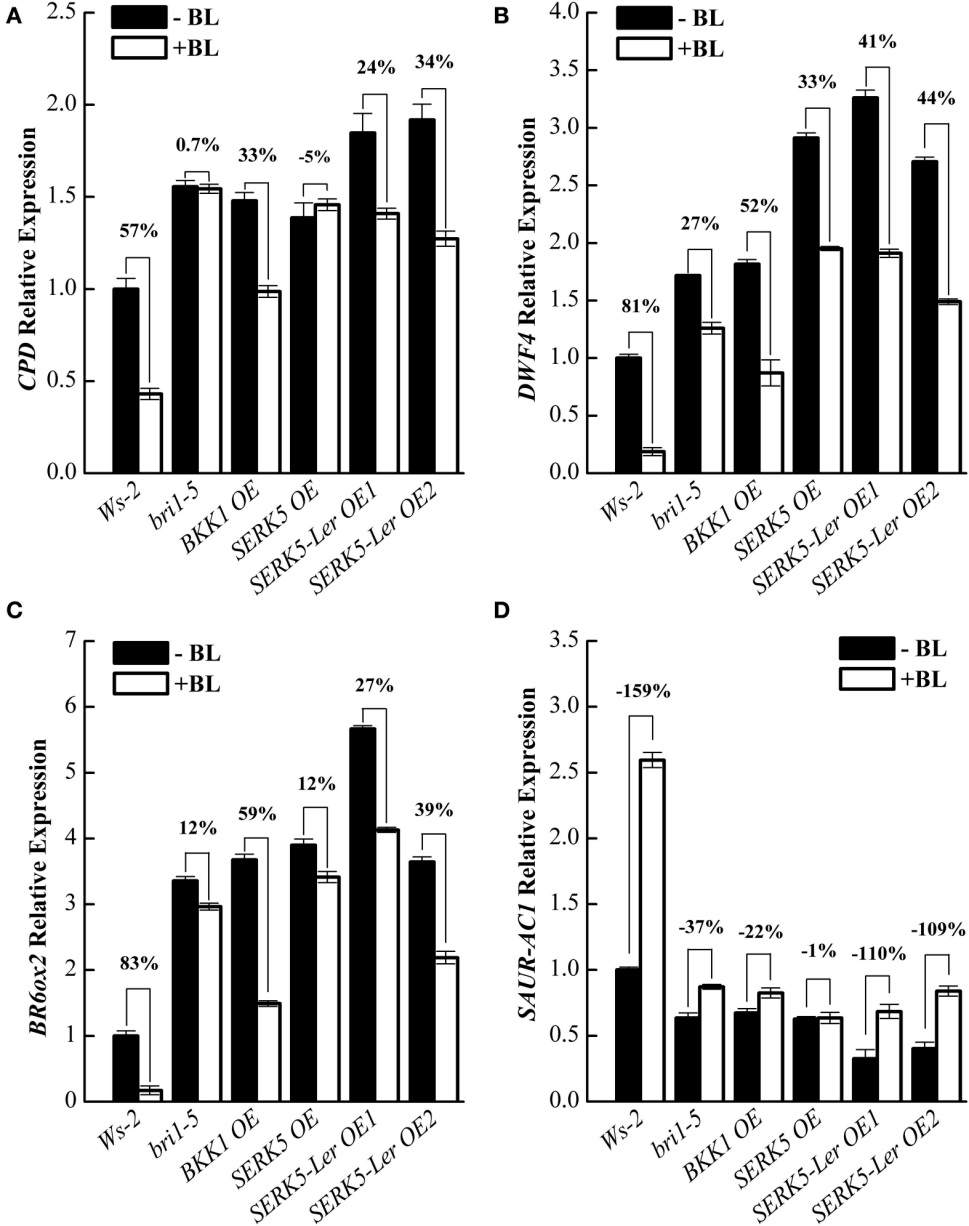
**Overexpression of ***SERK5***-L***er*** partially restores BR signaling in ***bri1-5***. (A–D)** 10-day-old seedlings grown on 1/2 MS medium were treated with or without 1 μM 24-epiBL for 3 h. Relative expression levels of *CPD, DWF4, BR6ox2*, and *SAUR-AC1* was examined by qRT-PCR. Overexpression of *SERK5*-L*er* partially recovers the sensitivity of BR-responsive genes in *bri1-5* upon BL treatment. *ACTIN2* was used as the reference gene. Values are means ± SE (*n* = 3). Experiments were repeated three times with similar results.

To further validate that *SERK5*-L*er* is truly involved in BR signaling, quantitative RT-PCR (qRT-PCR) experiments were performed to examine the expression levels of BR responsive genes such as *CPD, DWF4, BR6ox2*, and *SAUR-AC1* in wild-type and transgenic plants, when treated with or without BL. In WT plants, upon BL treatments, the expression of *CPD, DWF4*, and *BR6ox2* decreased 57%, 81%, and 83%, respectively, and the expression of *SAUR-AC1* increased 159%. Whereas, *CPD, DWF4* and *BR6ox2* showed only 0.7%, 27%, and 12% expression decrease, and *SAURN-AC1* showed 37% expression increase in *bri-5* after BL treatment, indicating a hampered BR signaling in *bri1*-5. The expression levels of *CPD, DWF4*, and *BR6ox2* decreased 24%/34%, 41%/54%, and 27%/39%, respectively, and the expression of *SAUR-AC1* increased 110%/109% in two *SERK5*-L*er* OE lines, suggesting the impaired BR signaling in *bri1-5* was partly recovered. These BR-regulated genes exhibited similar response to BL treatments in *bri1-5* overexpressing *BKK1* compared to *SERK5*-L*er* OE lines. The overexpression of *SERK5* in *bri1-5* mutant led to identical responses to BL treatments with its genetic background, confirming non-function of *SERK5* in BR signaling (Figures 4A–D).

### Overexpressing *SERK5*-L*er* can partially rescue the root and hypocotyl phenotypes of *bak1-3 bkk1-1* and increase its sensitivity to BR

**Figure 5:**
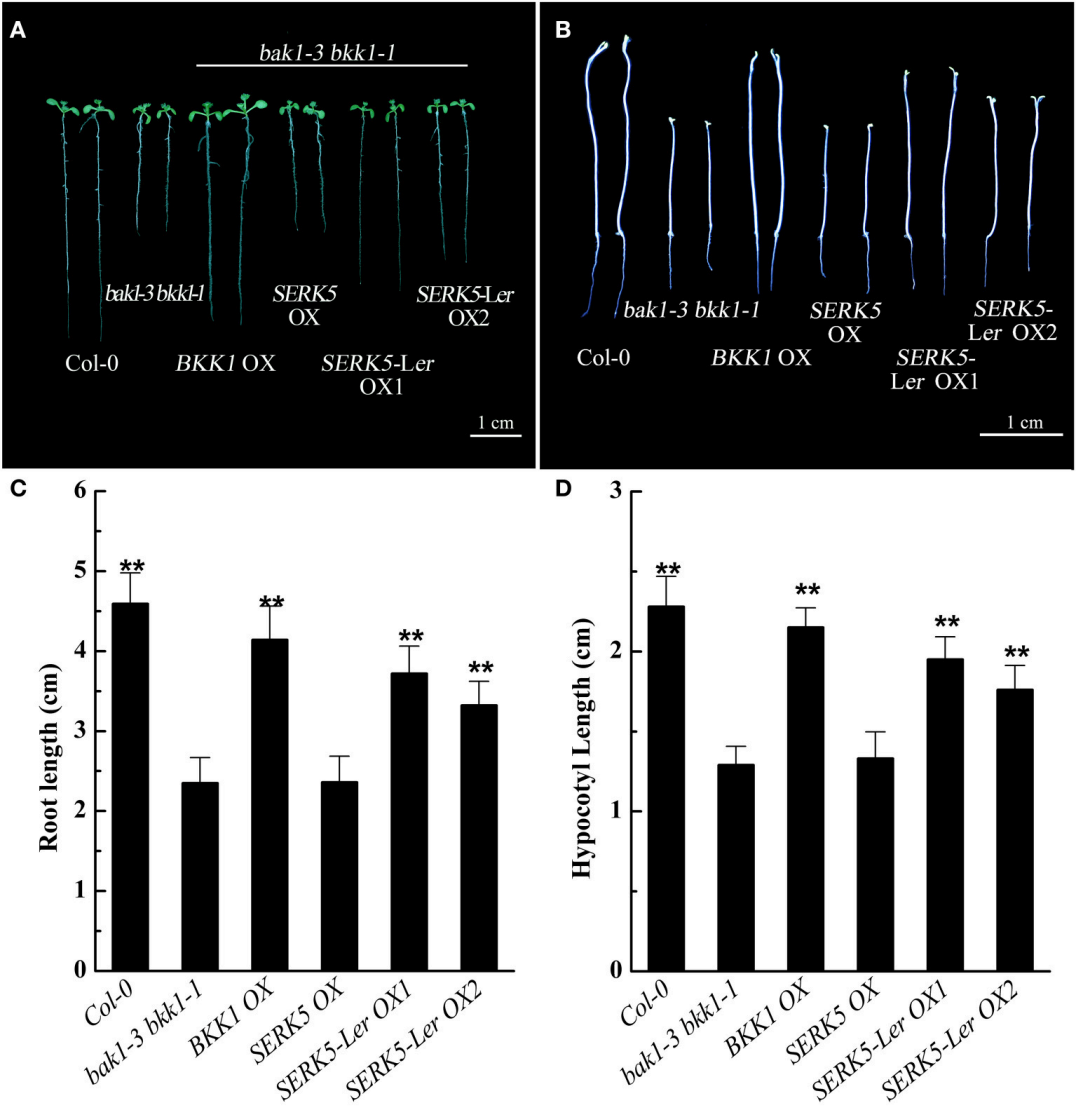
**Phenotypic analyses of ***bak1-3 bkk1-1*** overexpressing ***SERKs*****. **(A)** Overexpression of *SERK5*-L*er* elongates the root lengths of *bak1-3 bkk1-1*. 8-day-old seedlings grown on 1/2 MS medium in the light were presented. **(B)** Overexpression of *SERK5*-L*er* elongates the hypocotyl lengths of *bak1-3 bkk1-1*. 8-day-old seedlings grown on 1/2 MS medium in the dark were presented. **(C)** Statistic analyses of root lengths of the plants presented in **(A)**. **(D)** Statistic analyses of hypocotyl lengths of the plants presented in **(B)**. The data are shown as means ± standard deviation (SD) (*n* = 25). Student's *t*-test indicated the differences are statistically significant (^**^P < 0.01). Experiments were repeated three times with similar results.

**Figure 6:**
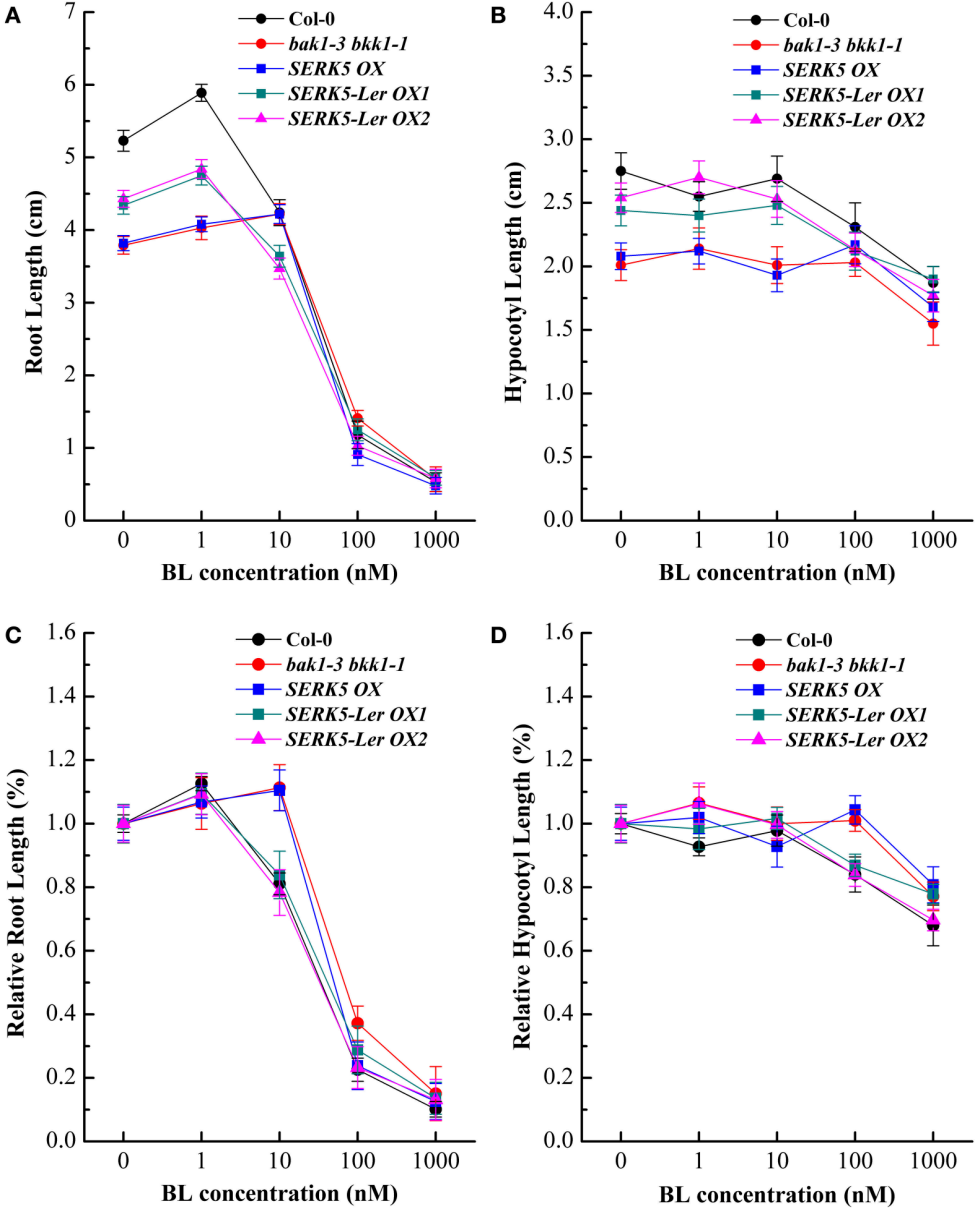
**Elevated expression of ***SERK5***-L***er*** in ***bak1-3 bkk1-1*** restore its sensitivity to BL**. The root and hypocotyl growth of 8-day-old seedlings grown on 1/2 MS medium containing different concentrations of 24-epiBL in the light and dark were analyzed. **(A)** Root lengths of the seedlings under 24-epiBL treatments. **(B)** Hypocotyl lengths of the seedlings under 24-epiBL treatments. **(C)** Relative root growth of the seedlings presented in **(A)**. Overexpression of *SERK5*-L*er* in *bak1-3 bkk1-1* restore its sensitivity when treated with 10 nM 24-epiBL. **(D)** Relative hypocotyl growth of the seedlings presented in **(C)**. Overexpression of *SERK5*-L*er* in *bak1-3 bkk1-1* restores its sensitivity when treated with 100 nM 24-epiBL. The data are shown as means ± standard deviation (SD) (*n* ≧ 40). Experiments were repeated five times with similar results.

Our previous studies indicated that the spontaneous cell death and BR defective phenotypes could be restored to wild-type-like by overexpressing *BAK1* or *BKK1* in null double mutant *bak1-4 bkk1-1* (He et al., 2007) and a weak double mutant *bak1-3 bkk1-1*. As the closest paralog of BKK1, SERK5-L*er* with an intact RD motif is postulated to function redundantly with BKK1. Transgenic plants of *bak1-3 bkk1-1* overexpressing *SERK5*-L*er, BKK1*, and *SERK5* fused to *FLAG* were generated, serving as a complementation assay (Figures 5A,B). Overexpression of the transgenes was confirmed by western-blotting analysis using anti-FLAG antibody (Figure S1B). As shown in Figure 5A, overexpression of *SERK5*-L*er* in *bak1-3 bkk1-1* (named as *SERK5*-L*er* OX) exhibited no strikingly different aerial phenotype but showed elongated root lengths compared with *bak1-3 bkk1-1* when grown in the light (Figures 5A,C). This complementation phenotype appeared to be weaker than *bak1-3 bkk1-1* overexpressing *BKK1* (named as *BKK1* OX). By contrast, overexpressing *SERK5* in *bak1-3 bkk1-1* (named as *SERK5* OX) caused no phenotypic alterations to *bak1-3 bkk1-1* (Figures 5A,B). When grown in the dark, the shorten hypocotyls, a typical BR mutant phenotype referred to “de-etiolation,” of *bak1-3 bkk1-1* was partially rescued by overexpressing *SERK5*-L*er* (Figures 5B,D), which, however, is not observed in *bri1-5* overexpressing *SERK5*-L*er* (Figures S2B,C). Comparing to *bak1-3 bkk1-1*, the root and hypocotyl lengths increased about 58%/41% and 51%/36%, respectively, in two *SERK5*-L*er* OX lines. Similarly, *BKK1* OX showed 76% and 67% increase in root and hypocotyl lengths, respectively, compared to *bak1-3 bkk1-1*. The root and hypocotyl growth was identical between *SERK5* OX and *bak1-3 bkk1-1*. Next, we performed root and hypocotyl growth inhibition assays to monitor the sensitivity of *SERK5*-L*er* OX lines in response to exogenously applied BL. The *SERK5*-L*er* OX lines appeared to restore the BR sensitivity in roots, when treated with 10 nM BL, similar to WT and distinct from its background *bak1-3 bkk1-1* and *SERK5* OX lines (Figures 6A,C). The hypocotyls of *SERK5*-L*er* OX lines also restored its BR sensitivity, when treated with 100 nM BL (Figures 6B,D). These results demonstrated that the expression of *SERK5*-L*er* effectively compensated the function of *BAK1* or *BKK1* in BR pathway in *bak1-3 bkk1-1*.

**Figure 7:**
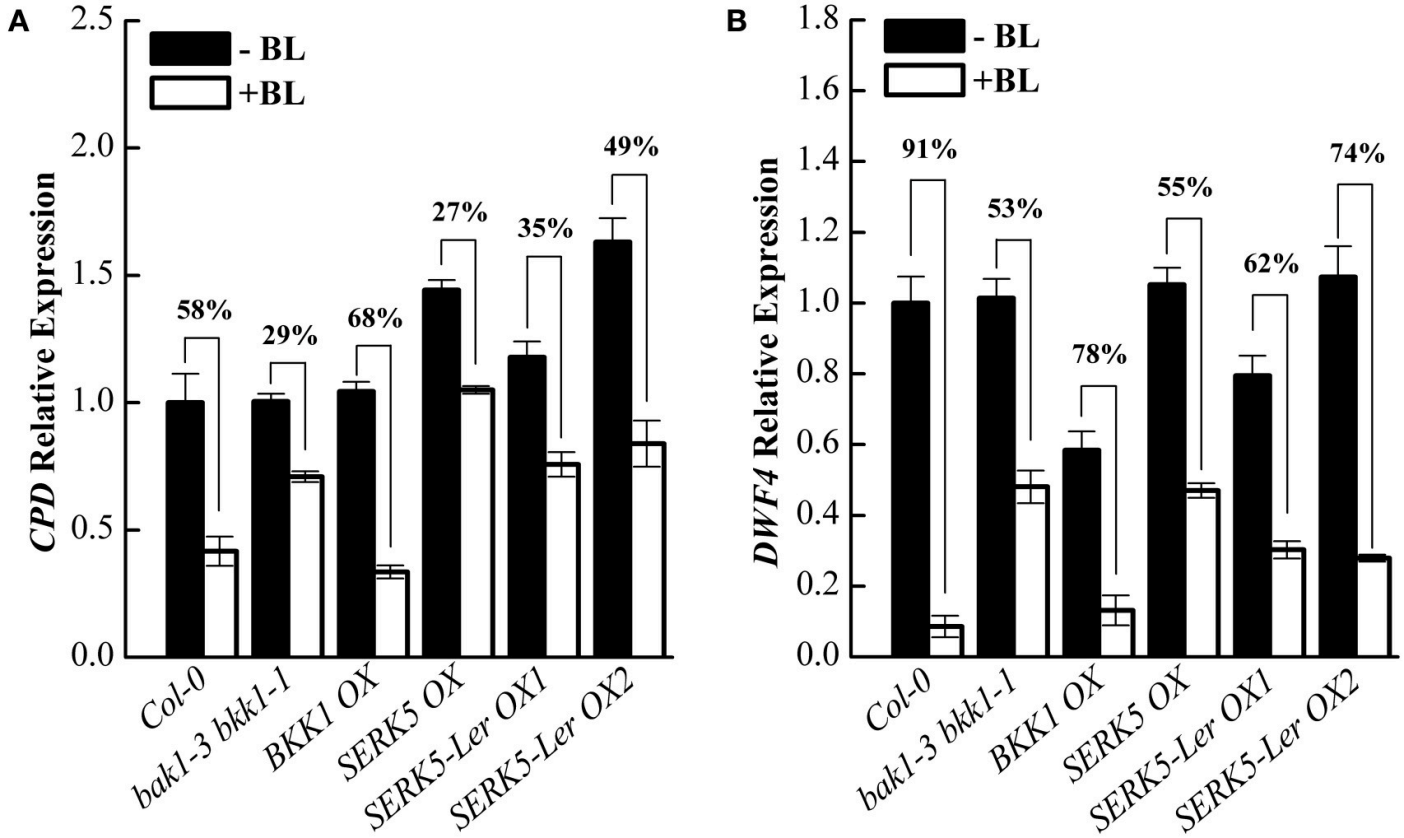
**Overexpression of ***SERK5***-L***er*** partially restores BR signaling in ***bak1-3 bkk1-1***. (A,B)** 10-day-old seedlings which grown on 1/2 MS medium were treated with or without 1 μM 24-epiBL for 3 h. Relative expression level of *CPD* and D*WF4* were measured by qRT-PCR. *bak1-3 bkk1-1* partially restores the sensitivity of BR-responsive genes to BL treatment by overexpressing *SERK5*-L*er*. *ACTIN2* was used as the reference gene. Values are means ± SE (*n* = 3). Experiments were repeated three times with similar results.

Subsequently, we analyzed the responses of BR-regulated genes *CPD* and *DWF4* in different genetic backgrounds. Upon BL treatment, the expression levels of *CPD* and *DWF4* were decreased about 58% and 91% in Col-0 wild-type but merely 29% and 53% in *bak1-3 bkk1-1*, respectively (Figures 7A,B). When treated with BL, the expression levels of *CPD* and *DWF4* decreased about 35%/49% and 62%/74% in two *SERK5*-L*er* OX lines, which were greater than those in *bak1-3 bkk1-1*, implicating an enhanced BR response in *bak1-3 bkk1-1* by overexpressing *SERK5*-L*er*.

Collectively, our genetic, physiological and molecular evidence verified that *SERK5*-L*er* but not *SERK5* plays a role in BR signaling.

### *SERK5*-L*er* interacts with BRI1 *in vivo*

**Figure 8:**
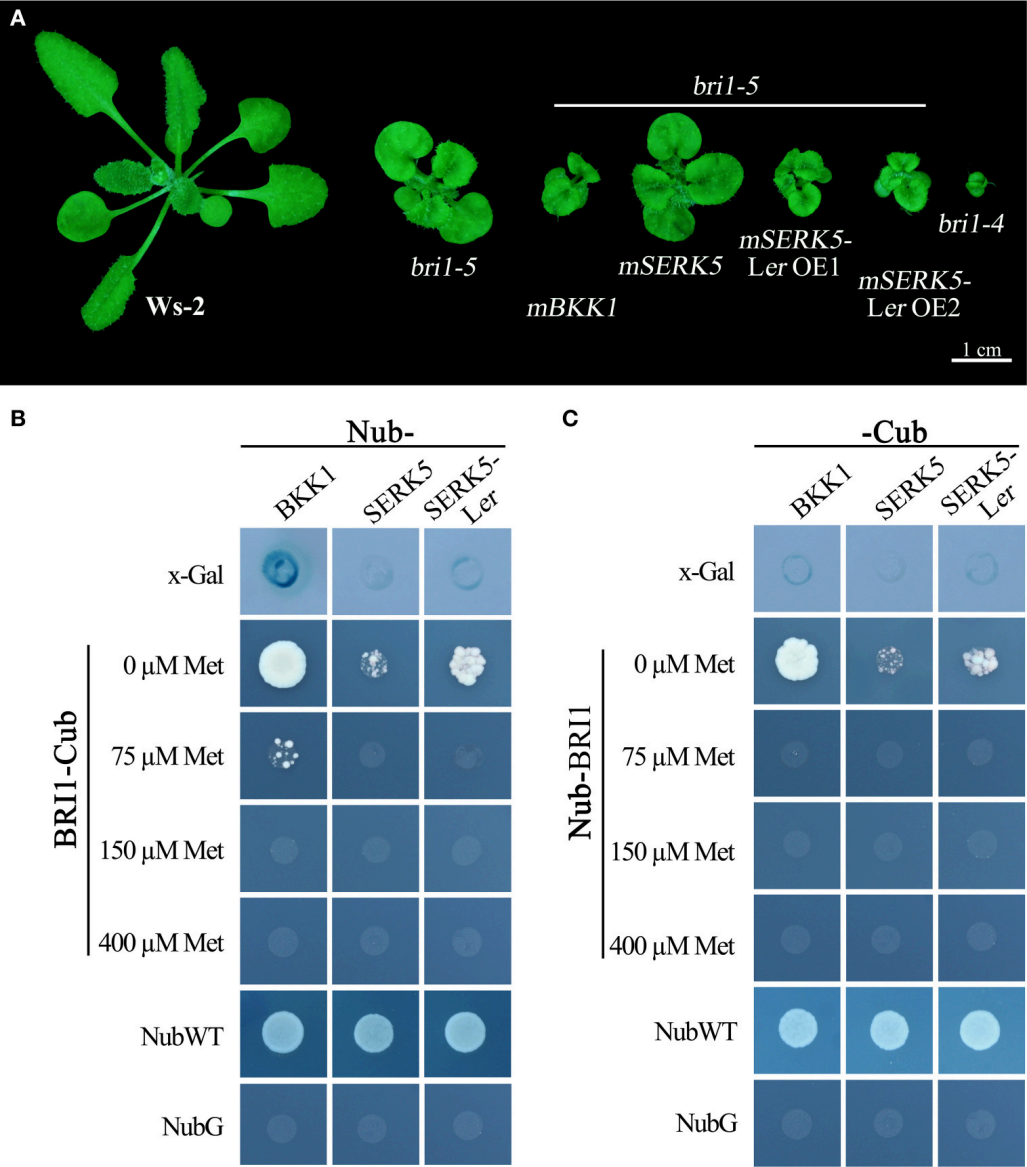
**SERK5-L***er*** interacts with BRI1**. **(A)** Overexpression of a kinase-inactive variant of SERK5-L*er* (K301E) led to a dominant-negative phenotype resembling *bri1-4*. Mating-based split ubiquitin system (mbSUS) to show BRI1-SERK5-L*er* interaction *in vivo*. Interaction was determined by the growth ability of mated yeast on selective medium (-AHLT) supplemented with 0, 75, 150, and 400 μM methionine. NubWT and NubG were used as positive and negative control, respectively. **(B)** *BRI1* was cloned as a -Cub fusion, and *SERK5*-L*er, BKK1*, and *SERK5* were cloned as a Nub- fusion. **(C)** *BRI1* was cloned as a Nub- fusion, and *SERK5*-L*er, BKK1*, and *SERK5* were cloned as a -Cub fusion.

BR molecules are perceived by ligand-binding receptor BRI1 and co-receptor SERKs. BR-induced formation of BRI1-SERKs heterodimer initiates downstream signaling cascades. If SERK5-L*er* truly participates in BR pathway, it is postulated to associate with BRI1. Our previous studies indicated that overexpression of a kinase-inactive version of SERK1 to SERK4 in *bri1-5* caused a typical dominant-negative phenotype which resembles a null *BRI1* mutant, *bri1-4* (He et al., 2007; Gou et al., 2012). Similarly, as shown in Figure 8A, overexpression of a kinase-dead form of *SERK5*-L*er* (K301E), not *SERK5* (K303E), in *bri1-5* resulted in typical dominant-negative phenotype, characterized by extreme dwarfism and severely compacted rosette leaves (Figure 8A). These results served as a piece of genetic evidence that SERK5-L*er* associates with BRI1 to mediate BR signaling *in planta* and that the kinase activity of *SERK5*-L*er* is crucial for its role in this process.

**Figure 9:**
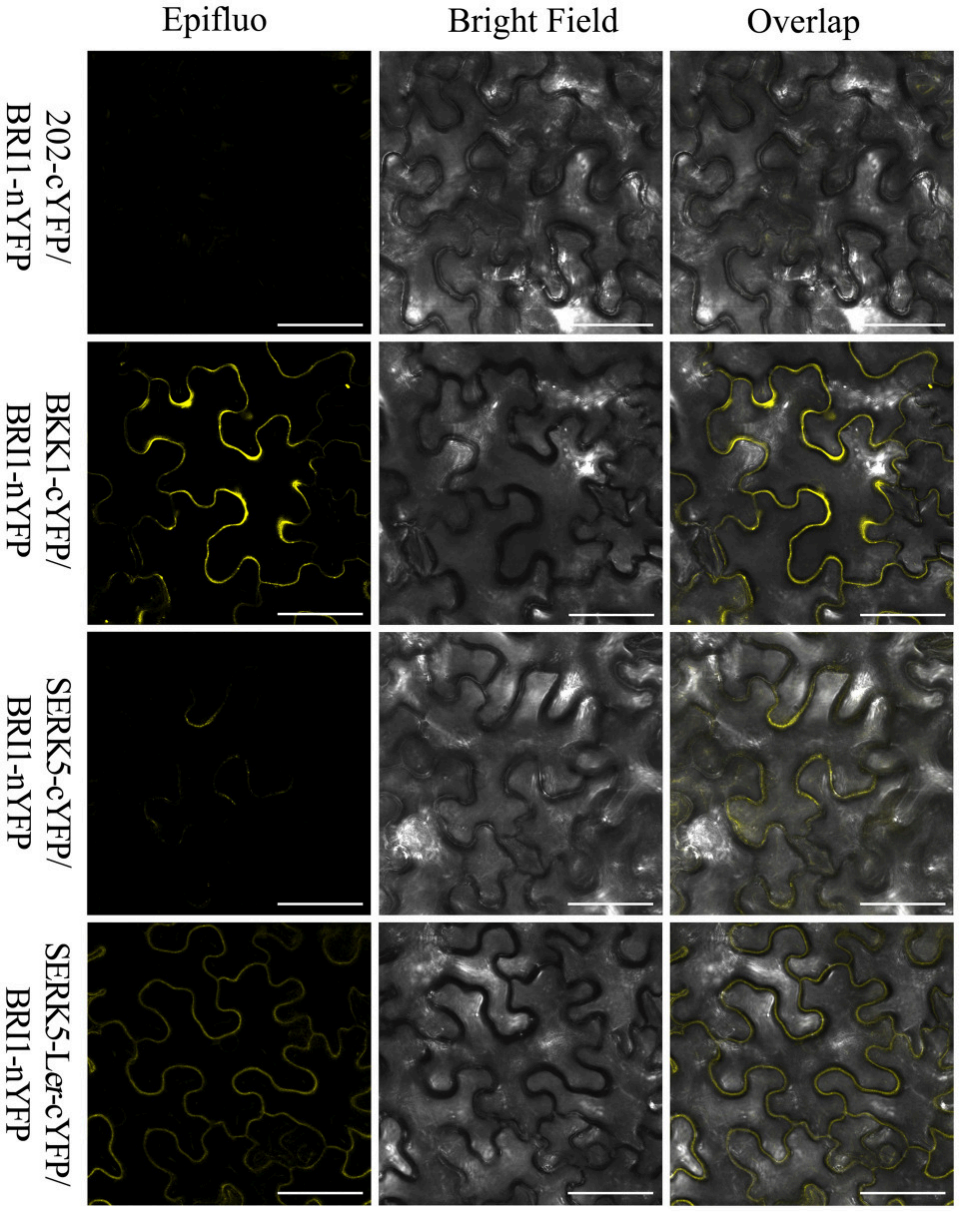
**BiFC assay to show the association of BRI1 and SERK5-L***er in planta*****. BRI1-nYFP was co-expressed with SERK5-L*er*-cYFP, BKK1-cYFP, and SERK5-cYFP in epidermal cells of *Nicotiana benthamiana*. Representative confocal images showing the YFP fluorescence signal indicate the physical interaction between BRI1 and SERK5-L*er*, BKK1, SERK5 at plasma membrane. BKK1 and SERK5-L*er* strongly interact with BRI1, while SERK5 only shows very weak binding with BRI1. Co-expression of 202-cYFP and BRI1-nYFP was used as a negative control. Similar results were obtained in more than three replicates. Scale bars = 50 μm.

Furthermore, a mating-based yeast split ubiquitin system (mbSUS) was used to examine the interaction between BRI1 and SERK5-L*er*/SERK5/BKK1. Consistent to the genetic data, the yeast two-hybrid result indicated that BRI1 strongly interacted with BKK1 (Figures 8B,C). Despite a slightly weaker interaction with BRI1 compared to BKK1-BRI1, SERK5-L*er* showed a stronger association with BRI1 in yeast system whereas SERK5 showed a much weaker interaction with BRI1. Moreover, the complex formed by BRI1 and SERK5-L*er* was verified by bimolecular fluorescence complementation (BiFC) assay. BRI1 was fused to the N-terminal half of YFP (BRI1-nYFP), while SERK5-L*er*, SERK5, and BKK1 were fused to the C-terminal half of YFP (SERK5-L*er*-cYFP, SERK5-cYFP, and BKK1-cYFP). Strong YFP fluorescence signals were detected at plasma membrane when *BRI1*-*nYFP* and *SERK5*-L*er*-*cYFP*/*BKK1*-*cYFP* were co-transformed in *Nicotiana benthamiana* epidermal cells, confirming the interaction between BRI1 and SERK5-L*er*/BKK1 *in planta*. Although detectable, the YFP fluorescence emitted by co-expression of *BRI1*-*nYFP* and *SERK5*-*cYFP* was very weak (Figure 9).

### Overexpression of *SERK5*-L*er* can delay the cell death phenotype of *bak1-3 bkk1-1*

**Figure 10:**
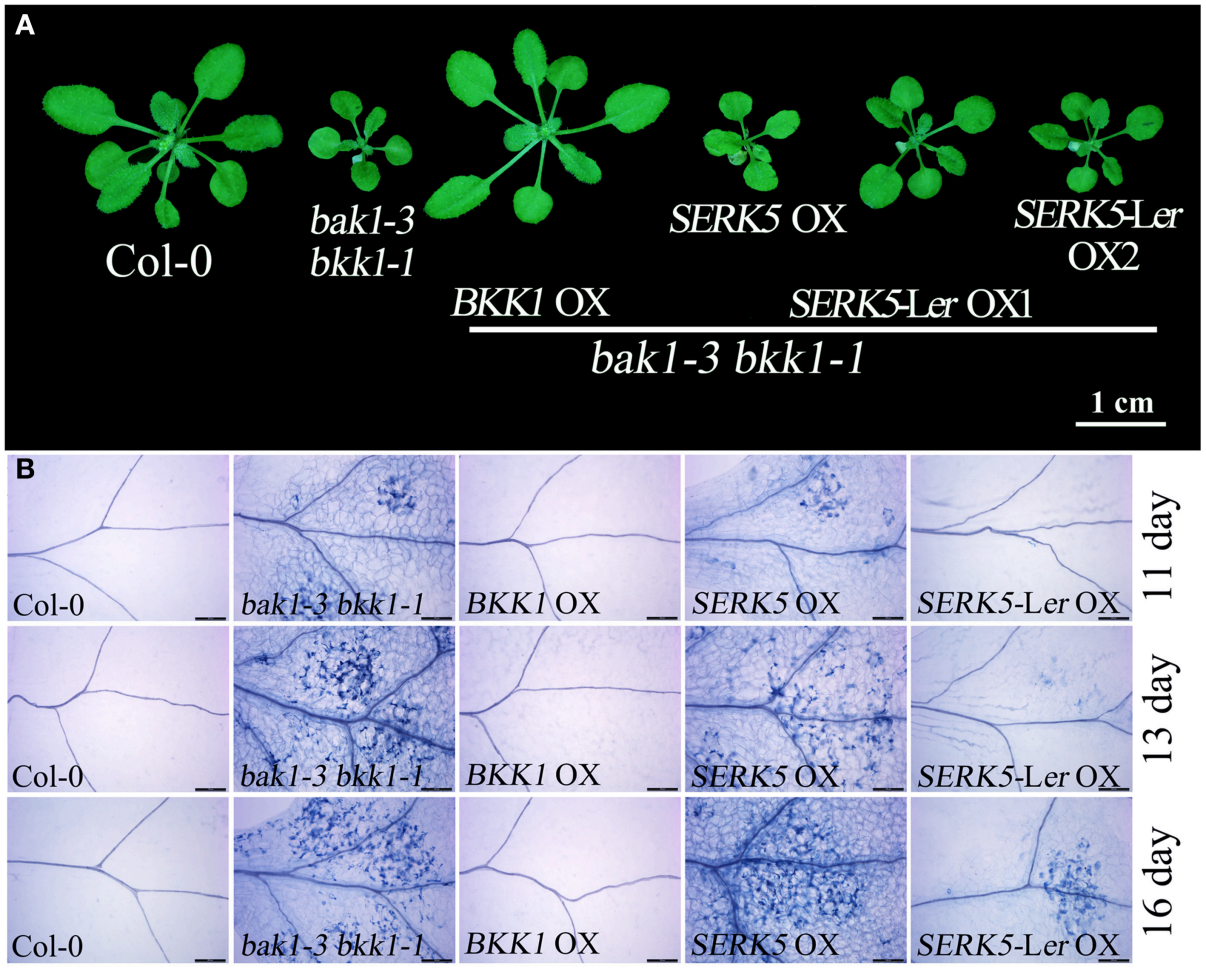
**Overexpression of ***SERK5***-L***er*** can delay the cell death in ***bak1-3 bkk1-1***. (A)** The aerial phenotype of 25-day-old seedlings. **(B)** The cell death was indicated by trypan-blue staining in the cotyledons of 11, 13, and 16-day-old seedlings grown at 22°C under long-day conditions (16 h light/8 h dark). Scale bars = 200 μm.

Our previous work has indicated that *BKK1* plays a central role in a BR-independent cell death control in addition to regulating BR signaling (He et al., 2007). Given that *SERK5*-L*er*, the closest paralog of *BKK1*, is indeed involved in BR pathway, it is logically hypothesized that *SERK5*-L*er* would also function in cell death control. As shown in Figure 10, overexpressing *BKK1* in *bak1-3 bkk1-1* completely suppressed its cell death symptom. However, overexpression of *SERK5*-L*er* failed to fully rescue the cell death phenotype of *bak1-3 bkk1-1* (Figure 10A). Subsequently, the cell death in the cotyledons of 11, 13, and 16-day-old seedlings grown on 1/2 MS medium were visualized by trypan-blue staining. The result showed that overexpression of *SERK5*-L*er* is capable of delaying the cell death symptom but is unable to completely suppress the cell death in *bak1-3 bkk1-1* (Figure 10B).

**Figure 11:**
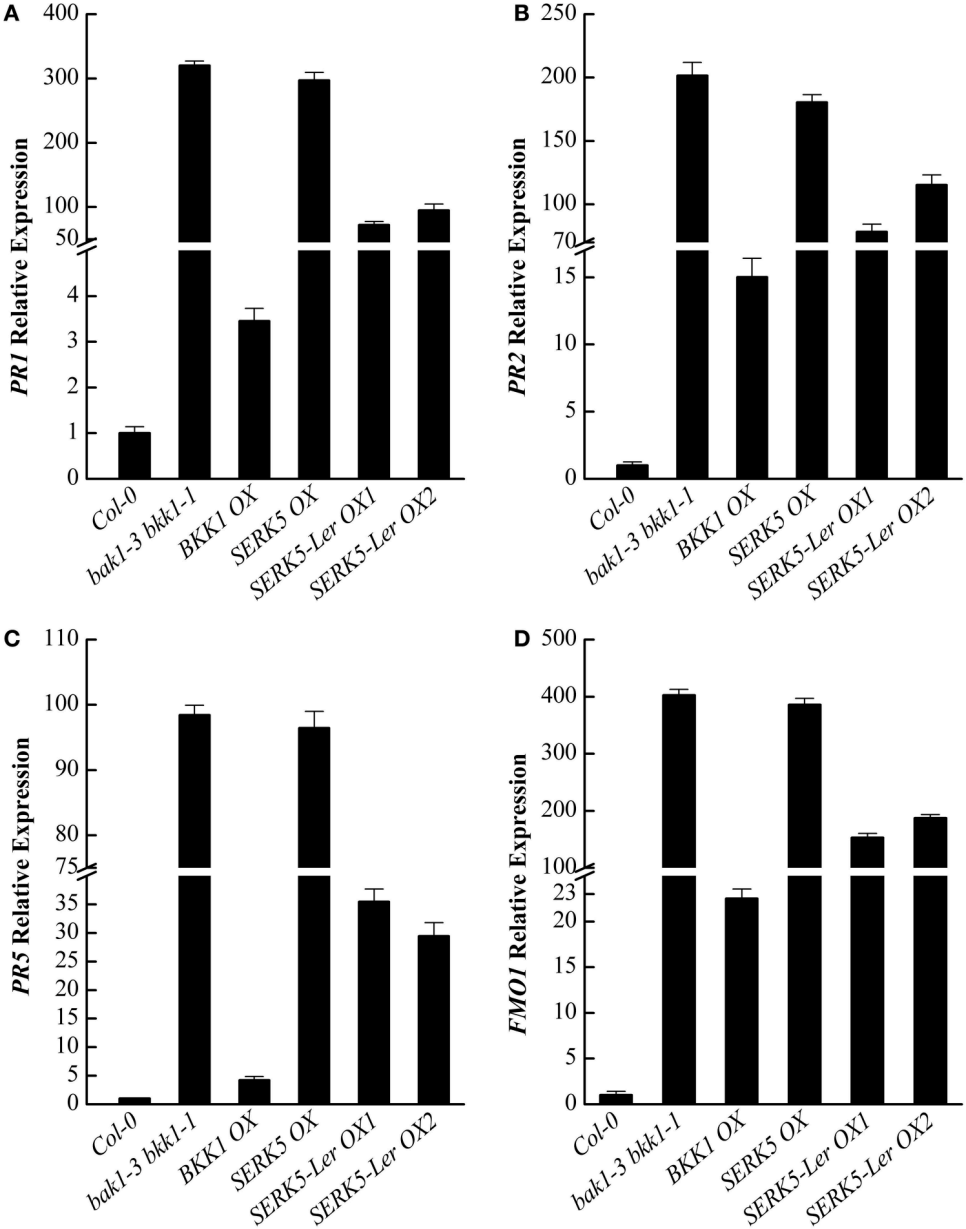
**qRT-PCR analysis of the ***PR1**, **PR2**, **PR5***, and ***FMO1*** gene expression levels in WT, ***bak1-3 bkk1-1*** and ***bak1-3 bkk1-1*** lines overexpressing ***SERKs*****. **(A–D)** Defense-related genes *PR1, PR2, PR5*, and senescence-related gene *FMO1* were down-regulated in *bak1-3 bkk1-1* when *SERK5*-L*er* is overexpressed.

It has been reported that some defense-related genes are highly up-regulated in *bak1 bkk1* (He et al., 2007; Yang et al., 2010). Therefore, the expression levels of pathogenesis-related genes *PR1, PR2, PR5*, and cell death marker gene *FMO1* in 13-day-old seedlings were analyzed. qPCR results indicated that the overexpression of *SERK5*-L*er* can slightly reduce the expression levels of defense and senesces-related genes in *bak1-3 bkk1-1* (Figures 11A–D), which is consistent with the phenotypic and trypan-blue staining results. Thus, our data suggested that *SERK5*-L*er* may play a role in cell death control, albeit less significantly than in BR signaling.

## Discussion

Perception of external stimuli and initiation of appropriate cellular responses are essential for plant growth and development, where RLKs act as crucial components. As a group of highly conserved *LRR-RLK* genes, *SERKs* are found to exist in all known plant genomes forming four major clusters including non-vascular *SERKs*, monocot *SERKs*, dicot *SERK1/2*, and dicot *SERK3/4* (Aan Den Toorn et al., 2015). It is indicated that the whole *SERK* family in *Arabidopsis* has originated from gene duplications followed by functional differentiation (Aan Den Toorn et al., 2015). The members of the SERK family in *Arabidopsis* have been identified to play multiple roles in a variety of developmental events via interacting with different ligand-binding RLKs. SERK1, SERK3/BAK1, and SERK4/BKK1 act as co-receptors for BR perception and signaling initiation (He et al., 2013). *SERK1* was reported to be involved in organ separation in flowers (Lewis et al., 2010). *SERK1* and *SERK2* also participate in male sporogenesis (Albrecht et al., 2005; Colcombet et al., 2005). BAK1 was found to form complexes with FLS2, EFR1, and BIR1, respectively, involved in plant immunity, and play overlapping role with BKK1 to regulate cell death (He et al., 2007; Kemmerling et al., 2007).

Despite numerous detailed functional analyses of *SERKs*, the biological roles of *SERK5* in *Arabidopsis* still remain blurred. In *Arabidopsis* ecotype Col-0, SERK5 bears an amino acid substitution of Arg (R) by Leu (L) within the critical arginine-aspartate (RD) motif in comparison with other four members in the SERK family (He et al., 2007). Therefore, it is interesting to explore whether the genomes of *Arabidopsis* ecotypes other than Col-0 contain *SERK5* genes encoding a LRR-RLK with intact RD motif that makes SERK5 a biologically functional regulator. To answer the question, we cloned *SERK5* cDNAs from L*er*, Col-0, Ws-2, Lan-0, and C24 accessions. Sequencing analysis indicated that *SERK5* in L*er* encodes a normal RD LRR-RLK (Figures 1A,C). The sequences of *SERK5* in Ws-2, Lan-0, and C24 contain a deletion of two nucleotides (TA), presumably resulting in a disruption of SERK5 protein translation (Figure 1B). Gene duplication serves as a key mechanism of expanding the gene number and diversifying the gene functions in plants and animals during evolution. Lehti-Shiu et al. reported that 30% of RLK family members are found in tandem repeats in *Arabidopsis*, suggesting higher frequency of RLK to be copied and clustered compared with other gene families (Lehti-Shiu et al., 2009). *BKK1* (At2G13790) and *SERK5* (At2G13800) are found as tandem repeats on chromosome 2 and share very high identity to each other, suggesting a recent duplication and high functional redundancy. As a result, one of these genes, *SERK5* may undergo low selection pressures during evolution, leading to a mutation in an extremely important region, RD motif, and frequent occurrences of nucleotide deletion. It is indicated that all plant SERKs, except SERK5 (in Col-0), contain RD motif (Aan Den Toorn et al., 2015). This result suggested that RD motif may be under purifying selection, the selection attempting to minimize amino acid change throughout evolution. Nevertheless, the possibility has not been ruled out that RD motif mutation, together with other SNPs, in *SERK5*-Col may confer novel functions yet to be characterized. Whether *SERK5* in Col-0 is a pseudogene needs further validation.

The natural genetic variations often shape the phenotypes of specific *Arabidopsis* strains. Numerous studies have described that different *Arabidopsis* populations vary in a number of traits (Weigel, 2012). A genetic method, quantitative trait locus (QTL) mapping, is widely utilized to identify loci responsible for specific traits in diverse *Arabidopsis* accessions. For instance, through QTL mapping in genetic combination of accessions displaying distinct trichome phenotypes, transcription factors ETC2 was identified to regulate trichome density (Weigel, 2012). Accession Gr-1 displays a low-trichome-number phenotype, which is modulated by *ETC2*, a major determining factor in trichome patterning. ETC2 in strain Can-0 contains an E19K substitution, leading to high trichome density (Hilscher et al., 2009). In addition to SERK5, other LRR-RLKs showing variations among *Arabidopsis* accessions are well documented, including non-RD kinase FLS2 and RD kinase ERECTA (ER). FLS2 perceives bacterial flagellin to initiate PAMP-triggered immunity (PTI) in plant. A polymorphism of FLS2 in Ws-0 accession leads to a stop-codon-mutation in the kinase domain, which makes Ws-0 insensitive to flg22, a 22 amino acid elicitor conserved in bacterial flagellin (Gómez-Gómez and Boller, 2002). ER functions as a key regulator in multiple physiological processes such as cell patterning, pathogenesis, and light and hormone signaling. It has been reported that several *Arabidopsis* accessions contain natural loss-of-function mutation in *ER* including L*er*, Hiroshima-1 (Hir-1) and Vancouver-0 (Van-0) (Van Zanten et al., 2009).

To assess whether *SERK5*-L*er*, like other *SERKs*, is involved in BR signaling, we overexpressed *SERK5*-L*er* in the *bri1-5* background. It has been validated to be an efficient approach to identify BR-related genes. When overexpressed, the genes involved in BR signaling pathway can either suppress or enhance the mutant phenotype of *bri1-5* (Ws-2 background) and the candidate genes can be isolated from the ecotypes other than Ws-2 (Li et al., 2002; He et al., 2007; Gou et al., 2012). Our results indicated that elevated expression of *SERK5*-L*er* but not *SERK5* can suppress the BR mutant phenotypes of *bri1-5* (Figure 2A). Furthermore, we introduced *SERK5*-L*er* into *bak1-3 bkk1-1*, which caused partially rescued root and hypocotyl lengths of *bak1-3 bkk1-1* and increased its sensitivity to exogenous BL (Figures 5, 6). The interaction of BRI1 and SERK5-L*er* was verified by different approaches (Figures 8, 9). Overexpression of a kinase death variant of *SERK5*-L*er* in *bri1-5* resulted in a null *bri1*-like phenotype, indicating SERK5-L*er* and BRI1 form complex that can be disrupted by occupation of BRI1 with highly accumulated kinase-inactive mSERK5-L*er*. In addition, yeast two-hybrid and BiFC analyses provided direct evidence that SERK5-L*er* is associated with BRI1 *in vivo* (Figures 8, 9). Our previous work showed that overexpression of *mSERK5* is unable to cause a dominant-negative phenotype in *bri1-5*, which is explicated by current results that SERK5 can barely associate with BRI1. Similar results were reported in an earlier study of allelic variation of *MYC1*. MYC1 is a bHLH transcription factor participating in trichome determination by associating with a scaffold protein, TTG1. MYC contains a P or A at amino acid position 189 in Col-0 and L*er*, respectively. In a yeast two-hybrid assay, MYC-Col interacts with TGG1 while the interaction is abolished when P189 is replaced by A. MYC-L*er* is not associated with TGG1, but an A189P substitution confers its interaction with TGG1 (Symonds et al., 2011). These results suggested that natural variation in MYC1 changes its biochemical nature and eventually causes distinct trichome density phenotype in Col-0 and L*er*. These results, together with our data, indicated that allelic variations may affect the function of a protein by altering the affinities with its partners when specific amino acids are changed.

It is worth noting that overexpressing *SERK5*-L*er* in *bri1-5* can partially rescue the BR defective phenotype of *bri1-5* by enlarging the size of rosette leaves. However, overexpression of *SERK5*-L*er* in *bri1-5* did not increase the lengths of petioles, roots, and hypocotyls, or recover the sensitivity of roots and hypocotyls of *bri1-5* to BL treatment (Figure 3, Figures S2, S3). This result is reminiscent of an earlier reports that overexpression of *SERK1, SERK2, BAK1*, and *BKK1* in *bri1-301*, a weak *bri1* mutant allele, led to partial restored rosette leaves, yet none of the transgenic lines is able to alter the hypocotyl and root phenotype (Albrecht et al., 2008). Interestingly, overexpression of *SERK5*-L*er* in *bak1-3 bkk1-1* appeared to rescue the BR defective phenotype by increasing the lengths of roots and hypocotyls of *bak1-3 bkk1-1*. These observations may suggest that BR signaling is more severely impaired in *bri1-5* mutant than that in *bak1-3 bkk1-1* double mutant (Gou et al., 2012). As a result, overexpression of *SERK5*-L*er* is insufficient to significantly rescue the root and hypocotyl phenotypes in *bri1-5*, yet it can effectively restore the root and hypocotyl phenotypes of *bak1-3 bkk1-1*.

Previous reports have demonstrated that *BKK1* is not only involved in the BR signaling, it also participated in the cell death control (He et al., 2007; Albrecht et al., 2008; Jeong et al., 2010). Our results revealed that *SERK5*-L*er* can only slightly rescue the cell death phenotype of *bak1-3 bkk1-1* when overexpressed. It suggested that *SERK5*-L*er* is preferentially engaged in BR signaling pathway than in cell death control pathway.

*BKK1* and *SERK5*-L*er* are not fully functionally redundant in a variety of signaling pathways. This divergence may be caused by the specific allelic variations in these two *SERKs*. The future studies will focus on the functional analyses of different amino acid residues in addition to RD motif in BKK1 and SERK5-L*er*. This may provide novel insights into the biological significance of specific residues of BKK1 and SERK5-L*er* in regulating diverse signaling pathways. The further investigations on *BKK1* and *SERK5*-L*er* can also serve as a paradigm for better understanding of the detailed molecular mechanisms of gene duplications and mutations, non-RD/RD kinase conversion, biological consequences of polymorphisms, and differentiations in gene functionality during evolution.

### Conflict of interest statement

The authors declare that the research was conducted in the absence of any commercial or financial relationships that could be construed as a potential conflict of interest.

## Supplementary material

Figure S1 **Western blotting to confirm the expressed protein by transgenes in different genetic background by using FLAG antibody. (A)** Overexpression of *SERK5*-L*er* in *bri1-5*. **(B)** Overexpression of *SERK5*-L*er* in *bak1-3 bkk1-1*. **(C)** Overexpression of kinase-death forms of *SERK5*-L*er* in *bri1-5*.

Figure S2 **Overexpression of *SERK5*-L*er* in *bri1-5* is unable to rescue the root and hypocotyl phenotypes of *bri1-5*. (A)** Statistic analyses of the average petiole lengths of 30-day-old WT, *bri1-5* and *bri1-5* lines overexpressing *SERKs*. **(B,C)** Statistic analyses of root and hypocotyl lengths of 8-day-old seedlings grown on 1/2 MS medium in light and dark, respectively. Overexpression of *BKK1* can partly recue the petiole phenotype, but not the root and hypocotyl phenotypes of *bri1-5*. Overexpression of *SERK5*-L*er* recues none of the petiole, root and hypocotyl phenotypes of *bri1-5*. The data are shown as means ± standard deviation (SD) (*n* ≧ 40). Student's *t*-test indicated the differences are statistically significant (^***^*P* < 0.001, ^**^*P* < 0.01). Experiments were repeated three times with similar results.

Figure S3 **Overexpression of *SERK5*-L*er* in *bri1-5* is unable to enhance the sensitive of *bri1-5* to BL. (A)** Root growth analysis of 8-day-old seedlings grown on 1/2 MS medium containing different concentrations of 24-epiBL in the light. **(B)** Hypocotyl growth analysis of 8-day-old seedlings grown on 1/2 MS medium containing different concentrations of 24-epiBL in the dark. The data are shown as means ± standard deviation (SD) (*n* ≧ 40). Experiments were repeated three times with similar results.

Table S1 **Primers used for gene cloning, mutagenesis, real-time RT-PCR and 24 RT-PCR. 25**.
