# Supplementary figures and images for: Somatic embryogenesis receptor-like kinase 5 in the ecotype Landsberg erecta of Arabidopsis is a functional RD LRR-RLK in regulating brassinosteroid signaling and cell death control
Source: Front Plant Sci. 2015 Oct 15;6:852. doi: 10.3389/fpls.2015.00852 (PMC4606071; doi:10.3389/fpls.2015.00852)

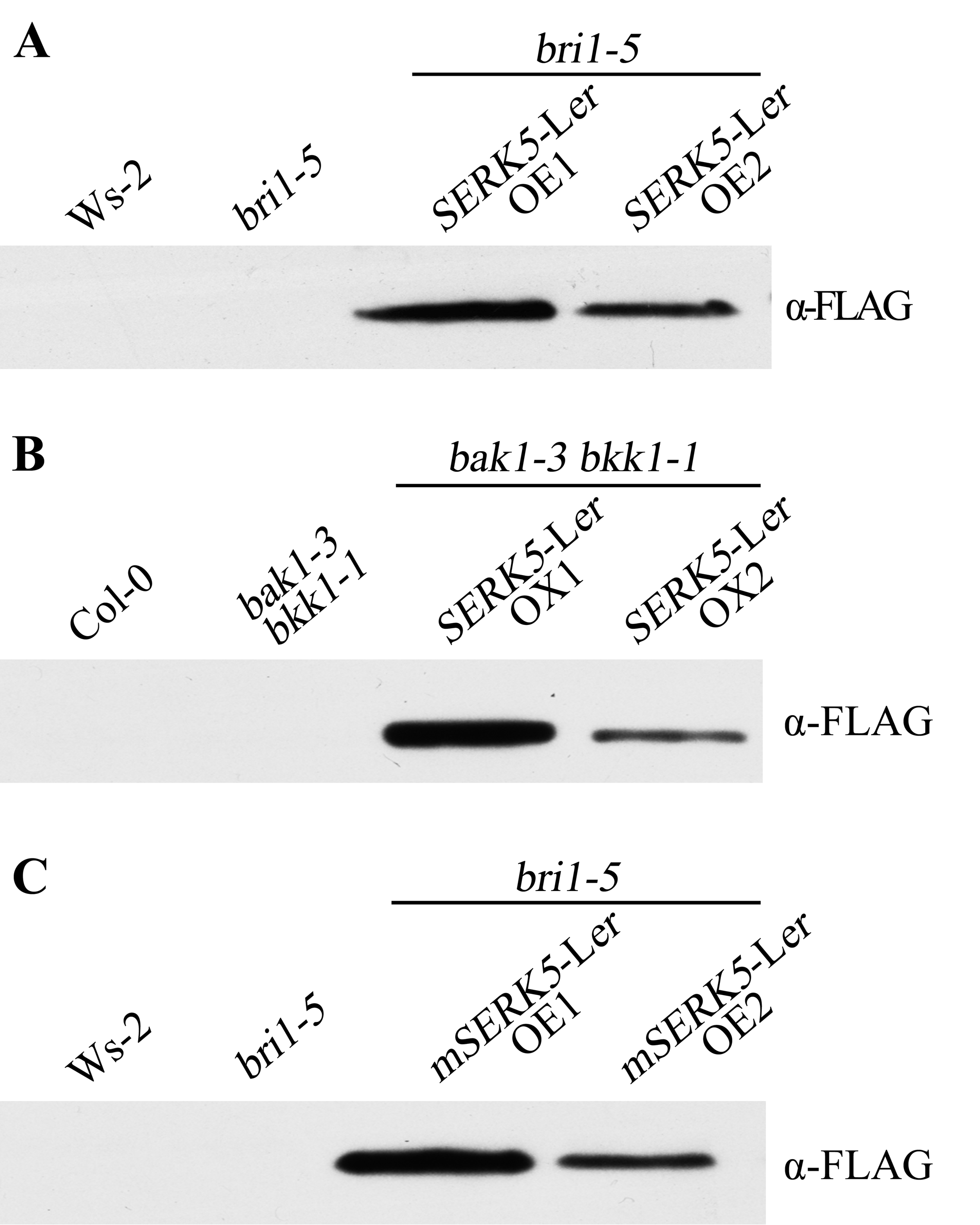

Supplement: Figure S1 — Western blotting to confirm the expressed protein by transgenes in different genetic background by using FLAG antibody. (A) Overexpression of SERK5-Ler in bri1-5. (B) Overexpression of SERK5-Ler in bak1-3 bkk1-1. (C) Overexpression of kinase-death forms of SERK5-Ler in bri1-5. [file Image1.TIF]

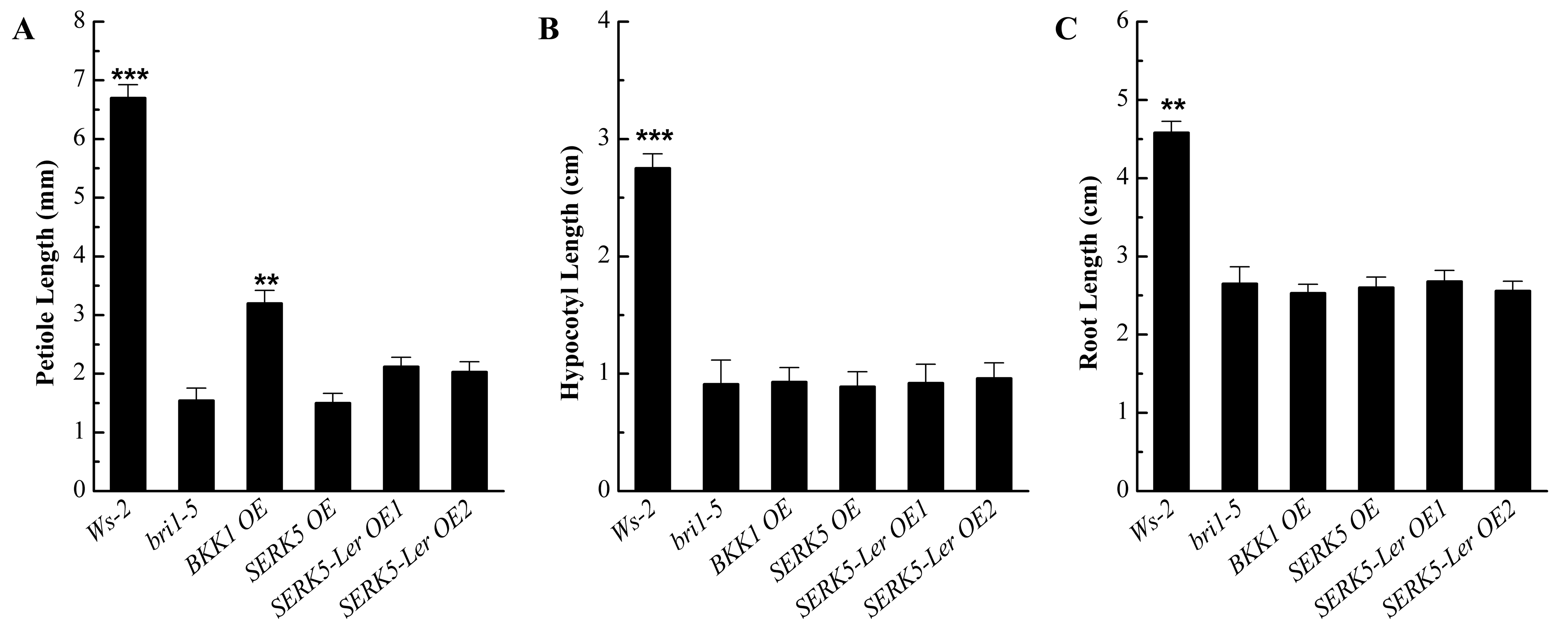

Supplement: Figure S2 — Overexpression of SERK5-Ler in bri1-5 is unable to rescue the root and hypocotyl phenotypes of bri1-5. (A) Statistic analyses of the average petiole lengths of 30-day-old WT, bri1-5 and bri1-5 lines overexpressing SERKs. (B,C) Statistic analyses of root and hypocotyl lengths of 8-day-old seedlings grown on 1/2 MS medium in light and dark, respectively. Overexpression of BKK1 can partly recue the petiole phenotype, but not the root and hypocotyl phenotypes of bri1-5. Overexpression of SERK5-Ler recues none of the petiole, root and hypocotyl phenotypes of bri1-5. The data are shown as means ± standard deviation (SD) (n ≧ 40). Student's t-test indicated the differences are statistically significant (***P < 0.001, **P < 0.01). Experiments were repeated three times with similar results. [file Image2.TIF]

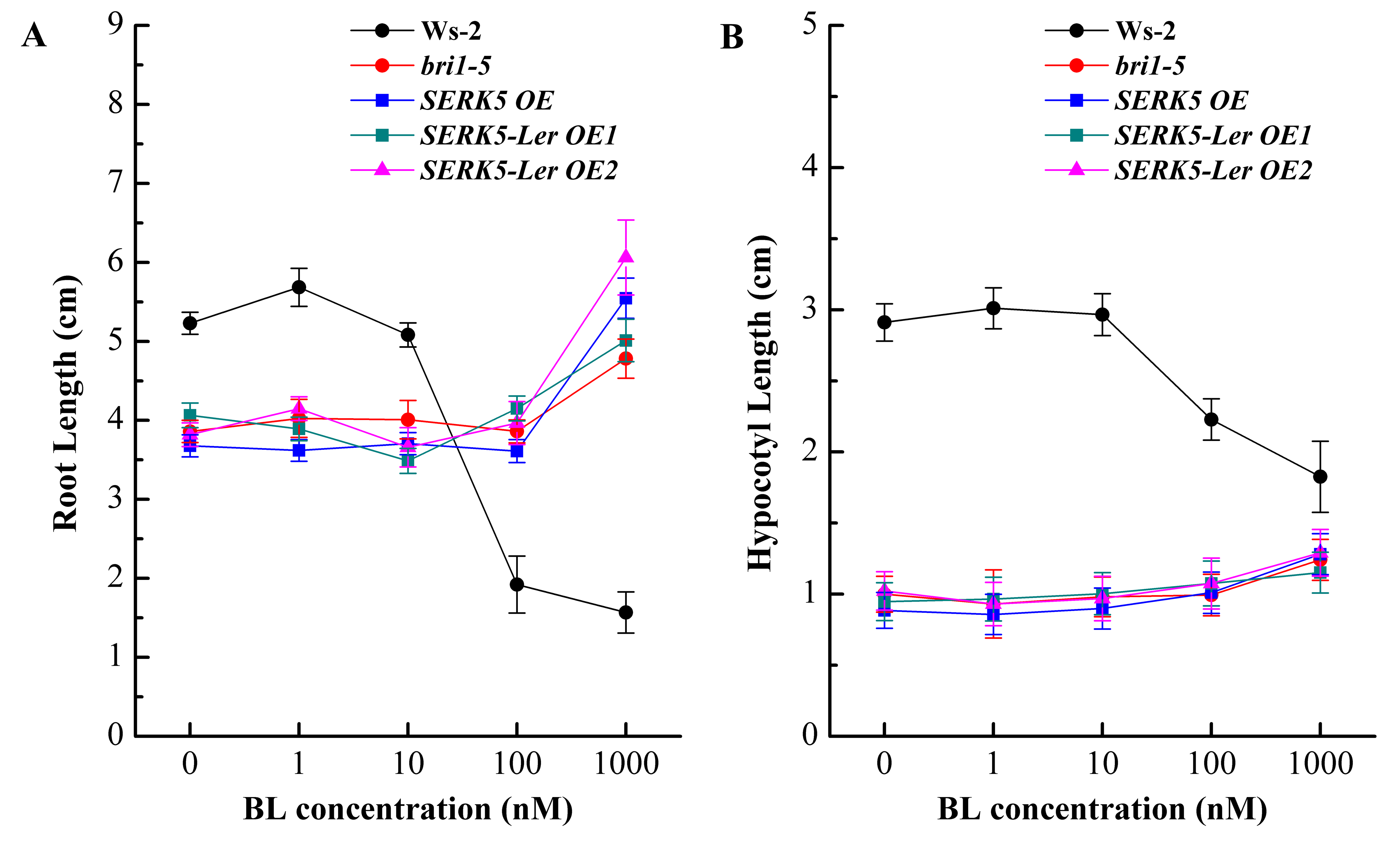

Supplement: Figure S3 — Overexpression of SERK5-Ler in bri1-5 is unable to enhance the sensitive of bri1-5 to BL. (A) Root growth analysis of 8-day-old seedlings grown on 1/2 MS medium containing different concentrations of 24-epiBL in the light. (B) Hypocotyl growth analysis of 8-day-old seedlings grown on 1/2 MS medium containing different concentrations of 24-epiBL in the dark. The data are shown as means ± standard deviation (SD) (n ≧ 40). Experiments were repeated three times with similar results. [file Image3.TIF]
