# Supplementary material for: Somatic embryogenesis receptor-like kinase 5 in the ecotype Landsberg erecta of Arabidopsis is a functional RD LRR-RLK in regulating brassinosteroid signaling and cell death control
Source: Front Plant Sci. 2015 Oct 15;6:852. doi: 10.3389/fpls.2015.00852 (PMC4606071; doi:10.3389/fpls.2015.00852)
Supplement: Table S1 — Primers used for gene cloning, mutagenesis, real-time RT-PCR and 24 RT-PCR. 25. [file Table1.DOCX]

**Table S1 |** Primers used for gene cloning, mutagenesis, real-time RT-PCR and RT-PCR

| **Primer name** | **Sequence** |
| --- | --- |
| **SERK5 cloning** | |
| SERK5C-F | AAAAAGCAGGCTTCATGGAACATGGATCATCCCGTG |
| SERK5C-R | AGAAAGCTGGGTCTCTTGGCCCCGAGGGGTAAT |
| SERK5G-F | AAAAAGCAGGCTTCTTCTCTTAGTCAATGGTGATTTCG |
| SERK5G-R | AGAAAGCTGGGTCGGGGTTTTGCTTCTTACAACTTC |
| **SERKs RT-PCR** | |
| SERK4CRT-F | ATGACAAGTTCAAAAATGGAACAAAG |
| SERK4CRT-R | TGGGTCTTCTTCAGCAGGTACAT |
| SERK5CRT-F | GCAGTTTATCATCAGGTGACCATA |
| SERK5CRT-R | TCACAGCCACTAGAGTGTCATCAG |
| SERK5LRT-F | AAGCAGTTTAGGTGACCATGCAA |
| SERK5LRT-R | GCCTTTTCACAGCCACTAGAGTG |
| ACTIN2RT-R | CAGTGGTCGTACAACCGGTATTG |
| ACTIN2RT-F | TGCTGTGATTTCTTTGCTCATACG |
| **site-directed mutagenesis** | |
| SERK4CKE-F | AATCTAGTGGCTGTCGAAAGGCTAAAAGAAG |
| SERK4CKE-R | CTTCTTTTAGCCTTTCGACAGCCACTAGATT |
| SERK5CKE-F | CACTCTAGTGGCTGTGGAACGGCTAAATGAAG |
| SERK5CKE-R | CTTCATTTAGCCGTTCCACAGCCACTAGAGTG |
| SERK5LKE-F | CACTCTAGTGGCTGTGGAAAGGCTAAAAGAAG |
| SERK5LKE-R | CTTCTTTTAGCCTTTCCACAGCCACTAGAGTG |
| **real-time RT-PCR** | |
| CPD qRT-F | GAGACGCTACGAGTGGCTAA |
| CPD qRT-R | GCATCTTTGAAGTGGTTTGGG |
| DWF4 qRT-F | CCACAACACTCGGTGACTTC |
| DWF4 qRT-R | CAGCTGATACGATCGTTGGTT |
| BR6ox2 qRT-F | CGGTTACCCGCAATCTATG |
| BR6ox2 qRT-R | TAAAGAAAGCAACGAGCCTC |
| SAUR-AC1 qRT-F | AGATATGTGGTGCCGGTTTC |
| SAUR-AC1 qRT-R | TTGTTAAGCCGCCCATTG |
| PR1 qRT-F | CATACACTCTGGTGGGCCTTA |
| PR1 qRT-R | CGCTAACCCACATGTTCACG |
| PR2 qRT-F | CGGGACGAGTGTGGAAAAC |
| PR2 qRT-R | ATAGCTTTCCCTGGCCTTCT |
| PR5 qRT-F | TCACCCACAGCACAGAGACA |
| PR5 qRT-R | CAATGCCGCTTGTGATGAAC |
| FMO1 qRT-F | CTCTTCTGCGTGCCGTAGTTT |
| FMO1 qRT-R | TCCCTTTATCCGCTTCCTCAA |
| ACTIN2 qRT-F | TGTGCCAATCTACGAGGGTTT |
| ACTIN2 qRT-R | TTTCCCGCTCTGCTGTTGT |
